# Chalcones inhibit firefly bioluminescence dependent on A and B-ring substitution pattern – a structure-activity study combined with molecular docking

**DOI:** 10.1080/14756366.2025.2509657

**Published:** 2025-06-09

**Authors:** Corinna Urmann, Michael Kirchinger, Herbert Riepl

**Affiliations:** a Organic-analytical Chemistry, Weihenstephan-Triesdorf University of Applied Sciences, Straubing, Germany; b TUM Campus Straubing for Biotechnology and Sustainability, Technical University of Munich, Straubing, Germany

**Keywords:** FLuc, Xanthohumol C, reporter gene assay, structure-activity study, false positives

## Abstract

Chalcones represent a privileged scaffold in medicinal chemistry, with pyranochalcones, featuring an additional chromane-like ring, identified as neurogenic and neuroprotective. Reporter gene assays, often used to study these and other effects, can produce false positives due to firefly luciferase stabilisation by inhibitors. The present study demonstrates that pyranochalcones inhibit firefly luciferase activity, with inhibition levels ranging from none to 100% and IC_50_ values of 7.82 µM to 92.99 µM. Furthermore, molecular docking offers potential structure-based explanations for the observed selectivity of compounds towards firefly luciferase inhibition. Even slight modifications in the molecular structure lead to significant changes in luciferase inhibition, underscoring the importance of these findings for understanding structure-activity relationships in reporter gene assays. Accordingly, caution is advised when using reporter gene assays based on firefly luciferase and pyranochalcones, as the IC_50_ values are within the range of concentrations commonly used in both *in vivo* and *in vitro* assays.

## Introduction

Chalcones are a widely distributed class of compounds in plants, serving as building blocks in the synthesis of flavonoids^1^ and exhibit a range of biological activities including anti-inflammatory, anti-cancer and anti-bacterial properties.^2^ Since chalcones, or 1,3-diphenyl-2-propen-1-ones can inhibit and activate a variety of targets and serve as a privileged scaffold in medicinal chemistry,^3^^,^^4^ this compound class is of significant interest to researchers. As previously demonstrated by our research group, some members of the pyranochalcone class exhibit neurogenic activity^5^^,^^6^, neuroprotective properties^5^ and the capacity to enhance the formation of neurons in the presence of endogenous inhibitors.^7^

This activity, being of outmost interest, deserves inspection of a broader panel of compounds. The demonstration of neurogenic activity is usually confirmed using immunostaining, a method which is lengthy and expensive. Following the development of a more convenient reporter gene assay dependent on a neuron-specific promoter,^8^ it was offered as an alternative. Accordingly, the neurogenic activity can be quantified using bioluminescence as a readout. Bioluminescence is a natural phenomenon that occurs as a result of an enzyme-catalyzed reaction involving the emission of visible light in a living organism. The firefly (*Photinus pyralis*) luciferin-luciferase (FLuc) system is the most widely used bioluminescent system and has been the subject of extensive investigation over several years.^9^ D-luciferin can be oxidised by FLuc to oxyluciferin, resulting in an emission wavelength range of 550 nm to 620 nm. Due to the sensitivity and suitability for both cell and enzyme assays, FLuc-based bioluminescence assays are highly preferred in (high throughput) screening approaches.^10^

However, there is a possibility of unexpected non-specific activation or inhibition of the enzyme, which could result in false positive or negative results. This is of particular importance in the context of reporter gene assays, when decisions will be made for future experimentation by using the resulting data.^10^

FLuc was inhibited by approximately 12% when tested against 360 000 compounds in a chemical library.^9^ These inhibitors have the ability to compete either the D-luciferin (e.g. benzothiazoles), or ATP (e.g. hydrazines), via non-competitive mechanisms (e.g. resveratrol), or via the formation of multisubstrate adduct inhibitors (e.g. ataluren).^9–11^ Several inhibitors of FLuc are already known, among others resveratrol,^11^ 3-[5-(2fluorophenyl)-1,2,4-oxadiazol-3-yl]benzoic acid,^12^ (E)-2-fluoro-4′methoxystilbene,^13^ N-pyridin-2-ylbenzamides,^14^ 2-phenylnaphthalenes,^15^ aryltriazoles,^16^ isoflavonoids^17^ and chalcones.^18^ Additionally, a stable complex can be formed between an inhibitor and FLuc, thereby preventing FLuc degradation.^19^ This is particularly crucial in the context of reporter gene assays, which are a valuable tool in cell culture research due to several advantages including simplicity, versatility, flexibility and reproducibility.^10^ Stabilising firefly luciferase in reporter gene assays that span multiple days would result in an accumulation of FLuc in cells, leading to elevated luciferase activity at the endpoint measurement.^20^ The consequence is, at a minimum, an elevated level of activity for the target being assayed, and in the most unfavourable scenario, a false positive result.

The interchangeable hydrogen atoms of chalcones allow the synthesis of a multitude of derivatives, which differ in the substitution pattern at both the A- and B-ring. In addition to hydroxyl and methoxy groups, there are also derivatives containing nonpolar alkyl chains such as prenyl groups or chromane-like rings.

**Figure 1.**
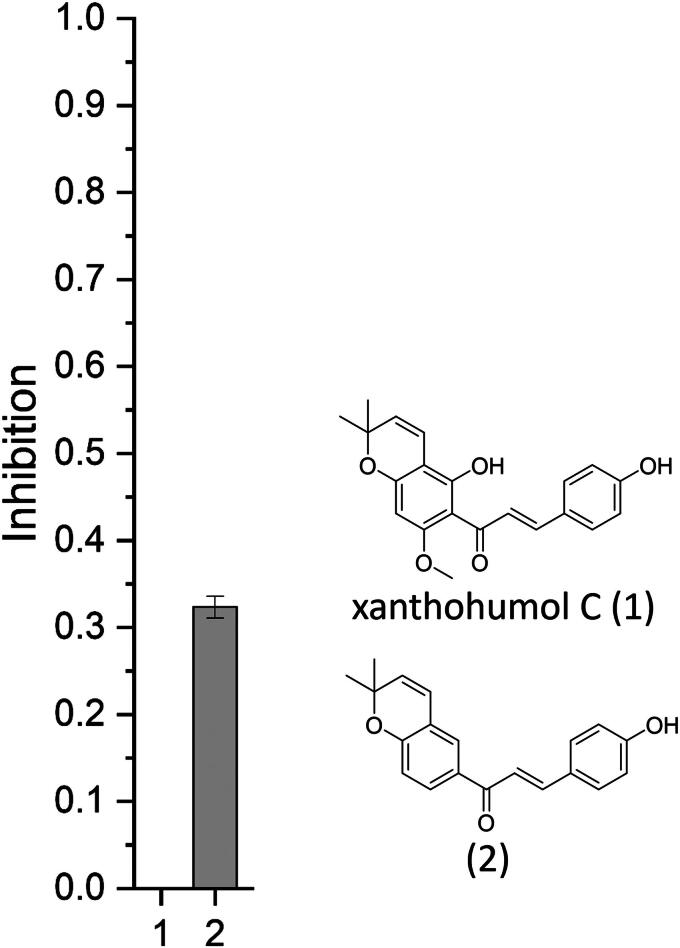
Inhibition (%) of Fluc by xanthohumol C (1) and compound 2 in a concentration of 10 µM (*n* = 3).

A structure-activity study was designed to distinguish the effects of diverse pyranochalcones on neurogenic activity. To facilitate the synthesis of this class of compounds, which is characterised by protection and deprotection steps, a simplified structure (**2**) (Figure 1) was used as the core structure. In contrast to the structure of xanthohumol C (**1**), which includes a substitution pattern at the A-ring with methoxy and hydroxyl groups as well as a hydroxyl group on the B-ring, the simplified structure is lacking theses groups at the A-ring. Since neurogenic activity is quantified through a reporter gene assay based on doublecortin with Fluc serving as the reporter enzyme,^8^ luciferase activity is routinely evaluated in our laboratory using purified recombinant firefly luciferase (*Photinus pyralis*). The findings of this study highlight that, for structurally similar compounds, interference with luciferase activity must be carefully considered to ensure accurate interpretation of reporter gene-based assays.

## Materials and methods

### General experimental procedures

NMR spectra were recorded using a JNM-ECS-400 (Jeol). Chemical shifts are given in ppm and multiplicity is abbreviated as follows: singlet (s), doublet (d), triplet (t), quartet (q), multiplet (m). A Shimadzu system (2xLC-20AD, SIL-20AC HT, CTO-20A, SPD-M20) with IT TOF as mass detector and equipped with a column (Phenomenex, Kinetex C_18_ 2.1 × 50 mm, 2.6 u) was used for ESI-HRMS-analytics using the following method: A (H_2_O + 0.1% formic acid) and B (MeCN + 0.1% formic acid); flow rate of 0.4 ml/min. Gradient elution: 00.00 − 5.35 min 35–95% B; then 5.35 − 6.35 min 95% B. Thin layer chromatography (TLC) was executed on TLC silica gel 60 F254 alumina sheets (Merck). Compounds were visualised under UV-light at λ = 254 nm, and λ = 360 nm and coloured compounds under daylight. Flash-chromatography was executed using a Puriflash 4250 (Interchim) with automatic program based on TLC Rƒ-values. Unless otherwise stated, chemicals for synthesis were purchased from Sigma-Aldrich (Taufkirchen, Germany) and VWR (Germany). The microwave irradiation was performed with a CEM DiscoverS class single mode synthesis system connected to a laptop PC running CEM synergy software to monitor the reaction. The temperature was controlled by an external infra-red sensor in the bottom of the cavity. When the target temperature was reached, the microwave system automatically started to count down the hold time. CEM 10 ml vials with snap-on caps were used for the reactions. Pressure was monitored by a sensor outside the snap-on caps. The upper pressure limit was set to 18 bar. The compounds were purified using preparative HPLC prior to use in the luciferase assay.

### Synthesis of compound 1 (xanthohumol C; ((E)-1–(7-hydroxy-5-methoxy-2,2-dimethyl-2H-chromen-6-yl)-3–(4-hydroxyphenyl)prop-2-en-1-one))

Xanthohumol was obtained by recrystallization from ­methanol/H_2_O (2/1) of the commercial prenylflavonoid-rich Xanthoflav^®^, kindly provided by Hallertauer Hopfenvera­rbeitungsgesellschaft m.b.H. The analytical data are in accordance with those published previously.^5^^,^^6^ To 1 mmol Xanthohumol, 6 ml of 1.4-dioxane and 1 mmol 2,3-dichloro-5,6-dicyano-1,4-benzoquinone (DDQ) were added and heated using microwave at a temperature 70 °C for 2.45 min. The reaction mixture was poured into 30 ml water and was extracted three times with 10 ml ethyl acetate. The organic layers were combined and washed with saturated sodium chloride solution and dried over MgSO_4_. The solvent was removed under reduced pressure and the orange product was purified by flash column chromatography. The analytical data are in agreement with previously published data.^5^^,^^6^

### Synthesis of different A rings

#### Synthesis of A-ring (1–(2,2-dimethyl-2H-chromen-6-yl)ethan-1-one) for compounds 2 and 8–20

Under nitrogen atmosphere, 1.0 mmol of 4-hydroxyacetophenone was dissolved in 5 ml of dry dimethylformamide. Then, 3.5 mmol potassium carbonate, 1.9 mmol potassium iodide, 0.1 mmol copper(II) chloride/copper(I) chloride and 1.3 mmol 3-chloro-3-methyl-1-butyne were added, and stirred at 65 °C for two hours. The reaction solution was transferred to 15 ml of water and extracted three times with 10 ml of ethyl acetate. The solvent was removed under reduced pressure. To increase the yield, the formation of the product was examined by means of gas chromatography and used directly in the next step. The crude product was dissolved in 15 ml diethylaniline under nitrogen atmosphere and heated using an oil bath at 190 °C for one hour. After cooling, 25 ml of ethyl acetate were added and washed three times with 20 ml of water each time. The solvent was removed under reduced pressure. The product was purified by column chromatography (n-hexane/ethyl acetate) on silica gel to give a light yellow oil with a yield of 75%.

^1^H-NMR (400 MHz, acetone-d_6_) δ (ppm) 1.43 (s, 6H, H-7′’ & H-8′’), 2.48 (s, 3H, CH_3_), 5.8 (d, 1H, J = 9.62 Hz, 5′’-H), 6.48 (d, 1H, J = 9.62 Hz, 4′’-H), 6.79 (d, 1H, J = 8.24 Hz, 5′-H), 7.68 (d, 1H, J = 2.29 Hz, 2′-H), 7.77 (dd, 1H, J = 10.53 Hz, 6′-H).^13^C-NMR (100 MHz, acetone-d_6_) δ (ppm) 25.91 (CH_3_), 28.04 (C-7′’ & C-8′’), 77.76 (C-6′’), 116.34 (C- 5′), 121.14 (C-3′), 121.91 (C-4′’), 127.32 (C-2′), 130.34 (C-6′), 131.07 (C-4′’), 131.79 (C-5′’), 157.56 (C-4′), 195.76 (C=O) TLC Rƒ: 0.46 (n-hexane/ethyl acetate 3/1).

### Synthesis of A-ring of compound 3–5

#### Protection of 2’,4’-Dihydroxyacetophenone

1 mmol 2′,4′-dihydroxyacetophenone was dissolved in 6 ml acetone, 7 mmol K_2_CO_3_ was added and the reagent mixture was heated to boiling point. Within one hour, 1.25 mmol MOM-Br was added dropwise and the reagent mixture was further heated for 4 h. After cooling to room temperature, K_2_CO_3_ was removed via filtration and the product was obtained as colourless oil after flash chromatography (TLC n-hexane/ethyl acetate 6/1 Rƒ 0.4). The yield was 64.5%.

#### O-Prenylation of protected acetophenone

1 mmol of the protected acetophenone was dissolved in 6 ml acetone, 7 mmol K_2_CO_3_ was added and the reagent mixture was heated to boiling point. Within one hour, 1.25 mmol 1-bromo-3-methyl-2-butene was added dropwise and the reagent mixture was further heated for 2 h. After cooling to room temperature, K_2_CO_3_ was removed via filtration and the product was obtained as colourless oil after flash chromatography (TLC n-hexane/ethyl acetate 6/1 Rƒ 0.34). The yield was 87.0%.

#### Rearrangement of prenyl group

The prenylated acetophenone was dissolved in *N,N*-dimethylaniline and heated to boiling point for 90 min. After cooling to room temperature the reagent mixture was acidified using 3 M HCl and extracted with ethyl acetate. The organic phase was washed three times with water, dried over Na_2_SO_4_. The product was obtained as colourless oil after flash chromatography (TLC n-hexane/ethyl acetate 6/1 Rƒ 0.29). The yield was 47.6%.

#### Deprotection of MOM-protection group

The acetophenone was then dissolved in 30 ml of methanol and heated to 60 °C. 0.5 ml of 3 M HCl were added and the reagent mixture was heated for 60 min to boiling point. The reagent mixture was poured to 30 ml of cold water and extracted with ethyl acetate, dried over Na_2_SO_4_. The product was obtained after flash-chromatography as a white solid. (TLC n-hexane/ethyl acetate 3/1 Rƒ 0.56). The yield was 62.0%.

#### Ring closure of prenylgroup to chromane like ring

The acetophenone was dissolved in 20 ml dry benzene with 200 µL dioxane. 1 eq DDQ was added and the reaction mixture heated to boiling point for 30 min. The reaction mixture was filtered and the product was obtained as yellow solid after purification using automated flash chromatography (TLC n-hexane/ethyl acetate 6/1 Rƒ 0.5). The yield was 65.0% of a light yellow solid.

#### Methylation of hydroxyl group –(1–(7-methoxy-2,2-dimethyl-2H-chromen-6-yl)ethan-1-one); synthesis of A-ring of compound 3

The acetophenone (1 mmol) was dissolved in 40 ml of a mixture of dichloromethane/water (3/2) and 1.50 mmol sodium hydroxide and 0.1 mmol tetrabutylammoniumchloride was added. To this reaction mixture, 1.20 mmol dimethylsulfate was added dropwise and the mixture was stirred for 24 h at room temperature. The reaction mixture was heated to ­boiling point for 30 min and extracted with dichloromethane. The solvent was removed under reduced pressure to obtain the colourless solid. (TLC n-hexane/ethyl acetate 2/1 Rƒ 0.25) The yield was 70.0%.

#### Synthesis of A-ring of compound 6

Under nitrogen atmosphere, 2.0 mmol of 4-aminoacetophenone was dissolved in 10 ml of toluene and mixed with 0.15 mmol of copper(II) chloride/copper(I) chloride and 3 mmol of 2-methyl-3-butin-2-ol. The reaction solution was stirred for 24 h at 120 °C. The solvent was removed by evaporation under reduced pressure. The product was obtained by purification (n-hexane/ethyl acetate) on silica gel as a light yellow oil with a yield of 42%. ^1^H-NMR (400 MHz, acetone-d_6_) δ (ppm) 1.32 (s, 6H, 7′’-H & 8′’-H), 2.37 (s, 3H, CH_3_), 5.52 (d, 1H, J = 9.86 Hz, 5′’-H), 5.99 (s, 1H, NH), 6.31 (d, 1H, J = 9.86 Hz, 4′’-H), 6.44 (d, 1H, J = 8.30 Hz, 5′-H), 7.48 (s, 1H, 2′-H), 7.59 (d, 1H, J = 8.30 Hz, 6′-H). ^13^C-NMR (100 MHz, acetone-d_6_) δ (ppm) 25.86 (CH_3_), 31.93 (C-7′’ & C-8′’), 53.26 (C-6′’), 112.11 (C- 5′), 118.70 (C-3′), 123.73 (C-4′’), 126.70 (C-4′), 128.18 (C-2′), 130.80 (C-6′), 131.59 (C-5′’), 149.01 (C-1′), 195.19 (C = O). TLC Rƒ: 0.21 (n-hexane/ethyl acetate 4/1).

#### Synthesis of A-ring compound 7 (1–(2,2-dimethyl-2-H-thiochromen-6-yl)ethan-1-one)

10.0 mmol thiophenol and 10.0 mmol 3,3-dimethylacrylic acid were dissolved in 0.3 ml piperidine. The reaction solution was heated to a temperature of 110 °C for 18 h. After cooling to room temperature the solution was poured into diluted hydrochloric acid and extracted three times with 15 ml ethyl acetate. The intermediate product was used without further processing steps. To close the ring, the reaction mixture was stirred for 30 min at 50 °C in polyphosphoric acid. The mixture was poured into water and extracted with ethyl acetate. The 6.8 mmol intermediate thus obtained was reacted with 3.4 mmol sodium borohydride in 20 ml dry ethanol for 15 min. The solvent was removed under reduced pressure and the residue was dissolved in dry benzene. After the addition of 0.35 mmol *p*-toluenesulfonic acid, the reaction mixture was heated to boiling point for 15 min. The solvent was removed under reduced pressure and the reaction mixture was purified by column chromatography. For acetylation, the product (6.1 mmol) was dissolved in 6 ml dry benzene and mixed with 9.5 mmol zinc chloride and heated to 80 °C. Subsequently, 7.4 mmol acetic anhydride were added in portions and the reaction mixture was heated for 30 min. After cooling to room temperature, 30 ml water was added and the product was extracted three times with 15 ml ethyl acetate each. The solvent was removed under reduced pressure. The product was purified by column chromatography (TLC: n-hexane/ethyl acetate 3/1 Rƒ 0.63) over silica gel to give a light yellow oil (yield 3%). ^1^H-NMR (400 MHz, acetone-d_6_) δ (ppm) 1.45 (s, 6H, 7′’-H & 8′’-H), 2.53 (s, 3H, CH_3_), 5.89 (d, 1H, J = 10.07 Hz, 5′’-H), 6.58 (d, 1H, J = 10.07 Hz, 4′’-H), 7.30 (d, 1H, J = 8.24 Hz, 5′-H), 7.74 (d, 1H, J = 8.24 Hz, 6′-H), 7.79 (s, 1H, 2′-H). ^13^C-NMR (100 MHz, acetone-d_6_) δ (ppm) 25.96 (CH_3_), 29.8 (C-7′’ & C-8′’), 41.93 (C-6′’), 126.11 (C-4′’), 127.05 (C-5′), 127.72 (C-6′), 127.93 (C-2′), 131.08 (C-3′), 134.87 (C-4′), 134.92 (C-5′’), 138.48 (C-1′), 196.36 (C = O).

### Aldol condensation of different A- and B-rings

If necessary the protection/deprotection of hydroxyl groups of aldehydes and benzaldehydes was performed according to the already published procedure.^6^ 1.0 mmol of actetophenone, if needed OH-groups protected with MOM, and the corresponding aldehyde, if needed protected with MOM, were dissolved in 13 ml methanol and 1.5 ml aqueous potassium hydroxide solution (50%) was added. The reaction mixture was heated to boiling point for 3 h. After cooling to room temperature, the reaction mixture was poured on 20 ml of water, acidified with hydrochloric acid and 3 times extracted with 15 ml of ethyl acetate. The solvent of organic layers was removed under reduced pressure. Deprotection: If needed, the MOM protected compound was dissolved in 10 ml methanol, five drops of hydrochloric acid (3 M) was added and the reaction mixture was heated for 60 min to 50 °C, followed by liquid-liquid extraction (15 ml water and 15 ml ethyl acetate) The solvent of organic layers was removed under reduced pressure and the product was obtained after purification on silica gel. The final products were further purified using preparative HPLC to obtain at least 95% purity of compounds.

#### (E)-1–(2,2dimethyl-2H-chromen-6-yl)-3–(4-hydroxyphenyl)prop-2-en-1-one (2)

1–(2,2-dimethyl-2H-chromen-6-yl)ethan-1-one; 4-(Methoxymethoxy)-benzaldehyde, yield: m = 103 mg (0.33 mmol, 61%) TLC: Rƒ 0.31 (*n-hexane*/ethyl acetate 3/1); ^1^H-NMR (400 MHz, acetone-d_6_) δ (ppm) 1.44 (s, 6H, 7′’-H & 8′’-H), 5.81 (d, 1H, J = 9.77 Hz, 5′’-H), 6.52 (d, 1H, J = 9.77 Hz, 4′’-H), 6.83 (d, 1H, J = 8.30 Hz, 5′-H), 6.91 (d, 2H, J = 8.30 Hz, 3-H & 5-H), 7.68 (m, α-H, β-H, 2-H & 6-H), 7.87 (s, 1H, 2′-H), 7.95 (d, 1H, J = 8.30 Hz, 6′-H). ^13^C-NMR (100 MHz, acetone-d_6_) δ (ppm) 28.47 (2xCH_3_), 78.19 (C-6′’), 116.64 (C-3 & C-5), 116.84 (C-5′), 119.54 (C-α), 121.70 (C-1), 122.41 (C-4′’), 127.79 (C-1′), 127.92 (C-2′), 130.17 (C-6′), 130.94 (C-5′’), 131.34 (C2 & C6), 132.16 (C-3′), 144.18 (C-β), 157.88 (C-4′), 160.57 (C-4), 187.93 (C = O). ESI-HRMS *m/z* calcd. for C_20_H_18_O_3_ [M + H]^+^ 307.1329 found 307.1332.

#### (E)-3–(4-hydroxyphenyl)-1–(7-methoxy-2,2-dimethyl-2H-chromen-6-yl)prop-2-en-1-one (3)

1–(7-methoxy-2,2-dimethyl-2H-chromen-6-yl)ethan-1-one; 4-(Methoxymethoxy)-benzaldehyde, yield: m = 40.1 mg (60%), TLC: Rƒ 0.11 (*n-hexane*/ethyl acetate 3/1) ^1^H-NMR (400 MHz, acetone-d_6_) δ (ppm) 1.44 (6H, s, 7′’-H & 8′’-H), 3.94 (3H, s, CH_3_), 5.66 (1H, d, *J* = 9.7 Hz, 5′’-H), 6.42 (1H, d, *J* = 9.9 Hz, 4′’-H), 6.51 (1H, s, 5′-H), 6.91 (2H, d, 3-H & 5-H), 7.43–7.58 (5H, m, 2-H & 6-H, 6′-H, α-H & β-H). ^13^C-NMR (100 MHz, acetone-d_6_) δ (ppm) 28.55 (2xCH_3_), 56.24 (OCH_3_), 78.16 (C-6′’), 100.81 (C-5′), 115.01 (C-3′), 116.71 (C-3 & C-5), 122.04 (C-4′’), 125.33 (C-α), 128.06 (C-1), 129.87& 129.72 (C-2′ & C-5′’), 131.04 (C-2 &C-7), 133.00 (C-2 & C-6), 142.15 (C-β), 158.63 (C-6), 160.36 (C-4), 161.25 (C-4′), 189.76 (C = O). ESI-HRMS m*/z* calcd. for C_21_H_20_O_4_ [M + H]^+^ 337.1434 found 337.1433.

#### (E)-1–(5-hydroxy-2,2-dimethyl-2H-chromen-6-yl)-3–(4-hydroxyphenyl)prop-2-en-1-one (4)

1–(5-(methoxymethoxy)-2,2-dimethyl-2H-chromen-6-yl)ethan-1-one; 4-(Methoxymethoxy)-benzaldehyde, yield: m = 11.6 mg (36%), TLC: Rƒ 0.34 (*n-hexane*/ethyl acetate 3/1) ^1^H-NMR (400 MHz, acetone-d_6_) δ (ppm) 1.42 (6H, s, 2xCH_3_), 5.70 (1H, d, *J* = 10.1 Hz, 5′’-H), 6.35 (1H, d, *J* = 8.9 Hz. 5′-H), 6.68 (1H, d, *J* = 10.1 Hz, 4′’-H), 6.95 − 6.87 (2H, d, 3-H & 5-H), 7.79 − 7.69 (3H, m, 2-H, 6-H, 6′-H), 7.72 (1H, d, β-H), 8.04 (1H, d, *J* = 8.9 Hz, α-H), 8.96 (1H, s, OH), 14.04 (1H, s, OH). ^13^C-NMR (100 MHz, acetone-d_6_) δ (ppm) 27.65 (2xCH_3_), 77.67 (C-6′’), 108.04 (C-5′’), 114.09 (C-3′), 115.48 (C-4′’), 115.98 (C-3 & C-5), 117.29 (C-6′), 126.74 (C-1), 131.12 (C0.2&C-6), 131.45 (C-α), 128.44 (C-5′’), 144.72 (C-β), 159.64 (C-2′), 160.46 (C-4), 160.92 (C-4′), 192.44 (C = O). ESI-HRMS *m/z* calcd. for C_20_H_18_O_4_ [M + H]^+^ 323.1278 found 323.1270.

#### (E)-1–(7-hydroxy-2,2-dimethyl-2H-chromen-6-yl)-3–(4-hydroxyphenyl)prop-2-en-1-one (5)

1–(7-hydroxy-2,2-dimethyl-2H-chromen-6-yl)ethan-1-one; 4-(Methoxymethoxy)-benzaldehyde, yield: m = 100.7 mg (32%), TLC: Rƒ 0.28 (*n-hexane*/ethyl acetate 3/1) ^1^H-NMR (400 MHz, acetone-d_6_) δ (ppm) 13.69 (1H, s), 9.00 (1H, s), 7.99 (1H, s), 7.85 (2H, d, *J* = 15.2 Hz, α-H & β-H), 7.73 (2H, d, J = 8.61 Hz, 2-H & 6-H), 6.94 (2H, d, J = 8.61 Hz, 3-H & 5-H), 6.44 (1H, d, *J* = 10.0 Hz, 4′’-H), 6.26 (1H, s, 5′-H), 5.72 (1H, d, *J* = 9.9 Hz, 5′’-H), 1.44 (6H, s, 2xCH_3_); ^13^C-NMR, (100 MHz, acetone-d_6_) δ (ppm) 28.73 (2xCH_3_), 78.76 (C-6′’), 104.76 (C-5′), 114.49 (C-3′), 116.82 (C-3 & C-5), 118.28 (C-4′’), 121.92 (C-α),. 127.57 (C-1′), 129.58 & 129.23 (C-2′ & C-5′’), 131.87 (C-2 & C-5), 145.47 (C-β), 161.15 (C-4&C-4′), 167.44 (C-6′), 192.97 (C = O). ESI-HRMS *m/z* calcd. for C_20_H_18_O_4_ [M + H]^+^ 323.1278 found 323.1285.

#### (E)-1–(2,2-dimethyl-1,2-dihydroquinolin-6-yl)-3–(4-hydroxyphenyl)prop-2-en-1-one (6)

1–(1,2-dihydro-2,2-dimethyl-6-quinolinyl)-ethanone; 4-(Methoxymethoxy)benzaldehyde yield: m = 46 mg (0.16 mmol, 36%) TLC: Rƒ 0.41 (*n-hexane*/ethyl acetate 3/1); ^1^H-NMR (400 MHz, acetone-d_6_) δ (ppm) 1.34 (s, 6H, 7′’-H & 8′’-H), 5.54 (d, 1H, J = 9.77 Hz, 5′’-H), 6.00 (s, 1H, NH), 6.36 (d, 1H, J = 9.77 Hz, 4′’-H), 6.49 (d, 1H, J = 8.30 Hz, 5′-H), 6.89 (d, 2H, J = 8.79 Hz, 3-H & 5-H), 7.63 (m, 4H, α−Η, β−Η, 2-H & 6-H), 7.70 (s, 1H, 2′-H), 7.77 (d, 1H J = 8.30 Hz, 6′-H). ^13^C-NMR (100 MHz, acetone-d_6_) δ (ppm) 31.98 (C-7′’& C-8′’), 53.47 (C-6′’), 112.32 (C-5′), 116.55 (C-3 & C-5), 118.94 (C-1), 119.96 (C-α), 123.81 (C-4′’), 127.63 (C-4′), 128.15 (C-2′), 128.38 (C-1), 130.98 (C-2 & C-6), 131.14 (C-6′), 131.56 (C-5′’), 142.54 (C-1′). 148.94 (C-β), 160.15 (C-4), 186.76 (C = O). ESI-HRMS *m/z* calcd. for C_20_H_19_NO_2_ [M + H]^+^ 306.1489 found 306.1503.

#### (E)-1–(2,2-dimethyl-2H-thiochromen-6-yl)-3–(4-methoxyphenyl)prop-2-en-1-one (7)

2,2-Dimethyl-6-acetyl-2H-thiochromene; 4-(Methoxymethoxy)-benzaldehyde yield: m = 69 mg (0.22 mmol, 46%), TLC: Rƒ 0.21 (*n-hexane*/ethyl acetate 3/1); ^1^H-NMR (400 MHz, acetone-d_6_) δ (ppm) 1.45 (s, 6H, 7′’-H & 8′’-H), 5.92 (d, 1H, J = 11.72 Hz, 5′’-H), 6.62 (d, 1H, J = 10.26 Hz, 4′’-H), 6.93 (d, 2H, J = 8.30 Hz, 3-H & 5-H), 7.25 (d, 1H, J = 8.30 Hz, 5′-H), 7.7 (m, 4H, α−Η, β−Η, 2-H & 6-H), 7.91 (d, 1H, J = 8.30 Hz, 6′-H), 7.98 (s, 1H, 2′-H). ^13^C-NMR (100 MHz, acetone-d_6_) δ (ppm) 30.23 (C-7′’& C-8′’), 42.46 (C-6′’), 116.70 (C-3 & C-5), 119.43 (C-α), 126.70 (C-4′’), 127.63 (C-1), 127.69 (C-5′), 128.43 (C-6′), 128.59 (C-2′), 131.49 (C-3′), 131.69 (C-2 & C-6), 135.35 (C-5′’), 136.55 (C-4′), 138.67 (C-1′), 144.82 (C-β), 160.76 (C-4), 188.51 (C = O). ESI-HRMS *m/z* calcd. for C_20_H_18_O_2_S [M + H]^+^ 323.1100 found 323.1105.

#### (E)-3–(4-aminophenyl)-1–(2,2-dimethyl-2H-chromen-6-yl)prop-2-en-1-one (8)

1–(7-methoxy-2,2-dimethyl-2H-chromen-6-yl)ethan-1-one; 4-aminobenzaldehyde, yield: m = 65 mg (52%), TLC: Rƒ: 0.11 (n-hexane/ethyl acetate 3/1) ^1^H-NMR (400 MHz, acetone-d_6_) δ (ppm) 1.44 (s, 6H, 7′’-H & 8′’-H), 5.27 (s, 2H, NH_2_), 5.81 (d, 1H, J = 10.26 Hz, 5′’-H), 6.51 (d, 1H, J = 9.77 Hz, 4′’-H), 6.71 (d, 2H, J = 8.30 Hz, 3-H & 5-H), 6.82 (d, 1H, J = 8.30 Hz, 5′-H), 7.54 (m, 3H, 2 & 6, α-H), 7.67 (m, 1H, β-H), 7.85 (s, 1H, 2′-H), 7.93 (d, 2H, J = 8.30 Hz, 6′-H). ^13^C-NMR (100 MHz, acetone-d_6_) δ (ppm) 28.46 (C-7′’ & C-8′’), 78.09 (C-6′’), 114.92 (C-3 & C-5), 116.76 (C-5′), 116.90 (C-α), 121.66 (C-4′), 122.50 (C-4), 127.78 (C-2′), 130.76 (C-6′), 131.35 (C-2 & C-6), 132.09 (C-5′’), 132.88 (C-3′), 145.13 (C-β), 152.20 (C-1), 157.62 (C-1′), 187.77 (C = O). ESI-HRMS *m/z* calcd. for C_20_H_19_NO_2_ [M + H]^+^ 306.1489 found 306.1487.

#### (E)-1–(2,2-dimethyl-2H-chromen-6-yl)-3–(4-(dimethylamino)phenyl)prop-2-en-1-one (9)

1–(7-methoxy-2,2-dimethyl-2H-chromen-6-yl)ethan-1-one; 4-(dimethylamino)benzaldehyde, yield: m = 375 mg (61%), TLC Rƒ 0.15 (n-hexane/ethyl acetate 3/1) ^1^H-NMR (400 MHz, acetone-d_6_) δ (ppm) 1.42 (s, 6H, 7′’-H & 8′’-H), 3.04 (s, 6H, 2xCH_3_), 5.81 (d, 1H, J = 9.77 Hz, 5′’-H), 6.51 (d, 1H, J = 9.77 Hz, 4′’-H), 6.76 (d, 2H, J = 8.79 Hz, 3-H & 5-H), 6.82 (d, 1H, J = 8.30 Hz, 5′-H), 7.65 (m, 4H, α-H, β-H, 2-H, 6-H), 7.85 (s, 1H, 2′-H), 7.93 (d, 1H, J = 8.55 Hz, 6′-H). ^13^C-NMR (100 MHz, acetone-d_6_) δ (ppm) 28.27 (C-7′’ & C-8′’), 39.94 (C-4–1 & C-4–2), 77.90 (C-6′’), 112.50 (C-3 & C-5), 116.58 (C-5′), 116.89 (C-α), 121.47 (C-1), 122.31 (C-4′’), 123.52 (C-3′), 127.58 (C-2′), 130.57 (C-6′), 130.91 (C-2 & C-6), 131.91 (C-5′’) 132.71 (C-1′), 144.74 (C-β), 152.84 (C-4), 157.43 (C-4′), 187.51 (C = O). ESI-HRMS *m/z* calcd. for C_22_H_23_NO_2_ [M + H]^+^ 334.1802 found 334.1775.

#### (E)-1–(2,2-dimethyl-2H-chromen-yl)-3–(4-methoxyphenyl)prop-2-en-1-one (10)

1–(7-methoxy-2,2-dimethyl-2H-chromen-6-yl)ethan-1-one; 4-methoxybenzaldehyde, yield: m = 120 mg (37%), TLC Rƒ 0.36 (n-hexane/ethyl acetate 3/1) ^1^H-NMR (400 MHz, acetone-d_6_) δ (ppm) 1.44 (s, 6H, H-7′’ & H-8′’), 3.85 (s, 3H, OCH_3_), 5.82 (d, 1H, J = 10.26 Hz, 5′’-H), 6.51 (d, 1H, J = 9.77 Hz, 4′’-H), 6.83 (d, 1H, J = 8.30 Hz, 5′-H), 7.00 (d, 2H, J = 8.79 Hz, H-3 & H-5), 7.72 (s, 2H, α-H, β-H), 7.76 (d, 2H, J = 8.30 Hz, H-2 & H-6), 7.88 (s, 1H, 2′-H), 7.96 (d, 1H; J = 8.30 Hz, 6′-H). ^13^C-NMR (100 MHz, acetone-d_6_) δ (ppm) 28.48 (C-7′’ & C-8′’), 55.74 (OCH_3_), 78.22 (C-6′’), 115.17 (C- 3 & C-5), 116.86 (C-5′), 120.26 (C-α), 121.72 (C-1), 122.41 (C-4′’), 127.96 (C-2′), 128.78 (C- 1′), 130.99 (C-6′), 131.12 (C-2 & C-6), 132.18 (C-5′’), 132.41 (C-3′), 143.79 (C-β), 157.93 (C- 4′), 162.53 (C-4), 187.85 (C = O). ESI-HRMS *m/z* calcd. for C_21_H_20_O_3_ [M + H]^+^ 321.1485 found 321.1475.

#### (E)-1–(2,2-dimethyl-2H-chromen-6-yl)-3-(p-tolyl)prop-2-en-1-one (11)

1–(7-methoxy-2,2-dimethyl-2H-chromen-6-yl)ethan-1-one; 4-methylbenzaldehyde, yield: m = 550 mg (69%), TLC: Rƒ 0.50 (n-hexane/ethyl acetate 3/1) ^1^H-NMR (400 MHz, acetone-d_6_) δ (ppm) 1.45 (s, 6H, 7′’-H & 8′’-H), 2.36 (s, 3H, CH_3_), 5.83 (d, 1H, J = 10.26 Hz, 5′’-H), 6.52 (d, 1H, J = 10.26 Hz, 4′’-H), 6.84 (d, 1H, J = 8.30 Hz, 5′-H), 7.27 (d, 2H, J = 8.30 Hz, 3-H & 5-H), 7.70 (d, 2H, J = 8.30 Hz, 2-H & 6-H), 7.74 (s, 1H, α-H), 7.79 (s, 1H, β-H), 7.89 (s, 1H, 2′-H), 7.97 (d, 1H, J = 8.30 Hz, 6′-H). ^13^C-NMR (100 MHz, acetone-d_6_) δ (ppm) 21.39 (C-4–1), 28.49 (C-7′’ & C-8′’), 78.27 (C-6′’), 116.91 (C-5′), 121.69 (C-α), 121.76 (C-3′), 122.37 (C-4′’), 128.06 (C-2′), 129.39 (C-2 & C-6), 130.41 (C-3 & C-5), 131.08 (C-6′), 132.22 (C-5′’), 132.26 (C-1′), 133.47 (C-4), 141.43 (C-1), 143,94 (C-β), 158.05 (C-4′), 187.93 (C = O). ESI-HRMS *m/z* calcd. for C_21_H_20_O_2_ [M + H]^+^ 305.1536 found 305.1524.

#### (E)-3–(4-chlorphenyl)-1–(2,2-dimethyl-2H-chromen-6-yl)prop-2-en-1-one (12)

1–(7-methoxy-2,2-dimethyl-2H-chromen-6-yl)ethan-1-one; 4-chlorobenzaldehyde, yield: m = 375 mg (63%), TLC Rƒ 0.42 (n-hexane/ethyl acetate 3/1) ^1^H-NMR (400 MHz, acetone-d_6_) δ (ppm) 1.27 (s, 6H, 7′’-H & 8′’-H), 5.98 (d, 1H, J = 10.26 Hz, 5′’-H), 6.82 (d, 1H, J = 10.26 Hz, 4′’-H), 6.98 (d, 1H, J = 8.30 Hz, 5′-H), 7.47 (d, 2H, J = 8.30 Hz, 3-H & 5-H), 7.71 (d, 2H, J = 8.30 Hz, 2-H & 6-H), 7.60 (s, 1H, α-H), 7.80 (s, 1H, β-H), 7.83 (s, 1H, 2′-H), 7.97 (d, 1H, J = 8.30 Hz, 6′-H). ^13^C-NMR (100 MHz, acetone-d_6_) δ (ppm) 28.20 (C-7′’& C-8′’), 45.47 (C-6′’), 115.70 (C-3 & C-5), 119.18 (C-α), 126.12 (C-4′’), 126.63 (C-1), 127.85 (C-5′), 127.93 (C-6′), 128.55 (C-2′), 131.47 (C-3′), 132.61 (C-2 & C-6), 134.35 (C-5′’), 137.35 (C-4′), 139.28 (C-1′), 145.80 (C-β), 161.96 (C-4), 189.37 (C = O). ESI-HRMS *m/z* calcd. for C_20_H_17_O_2_Cl [M + H]^+^ 325.0990 found 325.0934.

#### (E)-1–(2,2-dihmethyl-2H-chromen-6-yl)-3–(4-(trifluoromethyl)phenyl)prop-2-en-1-one (13)

1–(7-methoxy-2,2-dimethyl-2H-chromen-6-yl)ethan-1-one; 3-hydroxybenzaldehyde, yield: m = 250 mg (39%), TLC: Rƒ 0.35 (n-hexane/ethyl acetate 3/1) ^1^H-NMR (400 MHz, acetone-d_6_) δ (ppm) 1.38 (s, 6H, 7′’-H & 8′’-H), 5.04 (m, 2H, 3-H & 5-H), 5.77 (d, 1H, J = 9.77 Hz, 5′’-H), 6.42 (d, 1H, J = 9.77 Hz, 4′’-H), 6.66 (d, 1H, J = 8.79 Hz, 5′-H), 7.44 (d, 2H, J = 8.30 Hz, 2-H & 6-H), 7.55 (m, 3H, α-H, β-H, 2′-H), 7.65 (d, 1H, J = 8.55 Hz, 6′-H). ^13^C-NMR (100 MHz, acetone-d_6_) δ (ppm) 27.50 (C-7′’ & C-8′’), 41.85 (C-3), 49.28 (C-5), 77.42 (C-6′’), 115.87 (C-5′), 120.69 (C-3′), 121.41 (C-4′’), 124.68 (C-2), 124.72 (C-6), 127.06 (C-α), 129.05 (C-2′), 129.86 (C-4), 130.12 (C-6′), 131.37 (C-5′’), 144.44 (C-β), 157.31 (C-4′), 195.90 (C = O). ESI-HRMS *m/z* calcd. for C_21_H_17_F_3_O_2_ [M + H]^+^ 359.1253 found 359.1218.

#### (E)-1–(2,2-dimethyl-2H-chromen-6-yl)-3-phenylprop-2-en-1-one (14)

1–(7-methoxy-2,2-dimethyl-2H-chromen-6-yl)ethan-1-one; benzaldehyde, yield: m = 360 mg (67%), TLC: Rƒ 0.38 (n-hexane/ethyl acetate 3/1) ^1^H-NMR (400 MHz, acetone-d_6_) δ (ppm) 1.45 (s, 6H, 7′’-H & 8′’-H), 5.83 (d, 1H, J = 9.77 Hz, 5′’-H), 6.52 (d, 1H, J = 9.77 Hz, 4′’-H), 6.85 (d, 1H, J = 8.30 Hz, 5′-H), 7.44 (m, 3H, 3-H & 4-H & 5-H), 7.76 (s, 1H, β-H), 7.80 (m, 2H, 2-H & 6-H), 7.84 (s, 1H, α-H), 7.90 (s, 1H, 2′-H), 7.98 (d, 1H, J = 8.30 Hz, 6′- H). ^13^C-NMR (100 MHz, acetone-d_6_) δ (ppm) 25.50 (C-7′’ & C-8′’), 78.31 (C-6′’), 116.95 (C-5′), 121.79 (C- 4′), 122.36 (C-4′’), 122.77 (C-α), 128.09 (C-2′), 129.35 (C-2 & C-6), 129.75 (C-3 & C-5), 131.06 (C-4), 131.14 (C-6′), 132.17 (C-1′), 132.26 (C-5′’), 136.22 (C-1), 143.87 (C-β), 158.14 (C-4′), 187.93 (C = O). ESI-HRMS *m/z* calcd. for C_20_H_18_O_2_ [M + H]^+^ 291.1308 found 291.1298.

#### (E)-1–(2,2dimethyl-2H-chromen-6-yl)-3–(2-hydroxyphenyl)prop-2-en-1-one (15)

1–(7-methoxy-2,2-dimethyl-2H-chromen-6-yl)ethan-1-one; 2-(methoxymethoxy)benzaldehyde, yield: m = 360 mg (91%), TLC: Rƒ 0.21 (n-hexane/ethyl acetate 3/1) ^1^H-NMR (400 MHz, acetone-d_6_) δ (ppm) 1.43 (s, 6H, 7′’-H & 8′’-H), 5.80 (d, 1H, J = 9.86 Hz, 5′’-H), 6.50 (d, 1H, J = 9.86 Hz, 4′’-H), 6.82 (d, 1H, J = 8.30 Hz, 3-H), 6.90 (t, 1H, J = 7.27 Hz, 5-H), 6.96 (d, 1H, J = 8.30 Hz, 5′-H), 7.25 (t, 1H, J = 7.27 Hz, 4-H), 7.77 (d, 1H, J = 7.79 Hz, 6-H), 7.84 (m, 2H, α-H, 2′-H), 7.92 (d, 1H, J = 8.82 Hz, 6′-H), 8.13 (d, 1H, J = 15.57 Hz, β-H). ^13^C-NMR (100 MHz, acetone-d_6_) δ (ppm) 28.20 (C-7′’& C-8′’), 85.30 (C-6′’), 117.15 (C-3), 118.15 (C- 5′), 121.26 (C-5), 121.48 (C-α), 121.95 (C-3′), 122.87 (C-1), 122.96 (C-4′’), 126.15 (C-2′), 128.73 (C-6), 129.31 (C-4), 129.86 (C-6′), 130.23 (C-1′), 141.02 (C-β), 157.21 (C-2), 160.12 (C-4′), 189.71 (C = O). ESI-HRMS *m/z* calcd. for C_20_H_18_O_3_ [M + H]^+^ 307.1329 found 307.1316.

#### (E)-1–(2,2dimethyl-2H-chromen-6-yl)-3–(3-hydroxyphenyl)prop-2-en-1-one (16)

1–(7-methoxy-2,2-dimethyl-2H-chromen-6-yl)ethan-1-one; 3-(methoxymethoxy)benzaldehyde, yield m = 180 mg (70%), TLC: Rƒ 0.15 (n-hexane/ethyl acetate 3/1) ^1^H-NMR (400 MHz, acetone-d_6_) δ (ppm) 1.45 (s, 6H, 7′’-H & 8′’-H), 5.83 (d, 1H, J = 9.86 Hz, 5′’-H), 6.52 (d, 1H, J = 9.86 Hz, 4′’-H), 6.84 (d, 1H, J = 8.30 Hz, H-5′), 6.92 (m, 1H, H-2), 7.26 (m, 3H, H-4, H-5, H-6), 7.66 (d, 1H, J = 15.57 Hz, H-α), 7.77 (d, 1H, J = 15.57 Hz, H-β), 7.89 (s, 1H, H-2′), 7.97 (d, 1H, J = 10.90 Hz, H-6′). ^13^C-NMR (100 MHz, acetone-d_6_) δ (ppm) 28.50 (C-7′’& C-8′’), 78.30 (C-6′’), 115.76 (C-4), 116.95 (C- 2), 118.26 (C-5′), 120.68 (C-4′’), 121.69 (C-1′), 122.24 (C-2′), 122.37 (C-α), 122.71 (C-5′’), 122.94 (C-6), 128.08 (C-6′), 128.28 (C-5′’), 131.13 (C-5), 144.00 (C-β), 158.12 (C-3), 158.69 (C-4′), 187.95 (C = O). ESI-HRMS *m/z* calcd. for C_20_H_18_O_3_ [M + H]^+^ 307.1329 found 307.1338.

#### (E)-3–(3,4-dihydroxyphenyl)-1–(2,2-dimethyl-2H-chromen-6-yl)prop-2-en-1-one (17)

1–(7-methoxy-2,2-dimethyl-2H-chromen-6-yl)ethan-1-one; 3,4-bis(methoxymethoxy)benzaldehyde, yield: m = 320 mg (80%), TLC: Rƒ 0.43 (n-hexane/ethyl acetate 2/1) ^1^H-NMR (400 MHz, acetone-d_6_) δ (ppm) 1.44 (s, 6H, 7′’-H & 8′’-H), 5.81 (d, 1H, J = 9.86 Hz, 5′’-H), 6.51 (d, 1H, J = 9.86 Hz, 4′’-H), 6.83 (d, 1H, J = 8.82 Hz, 5-H), 6.89 (d, 1H, J = 7.79 Hz, 5′-H), 7.17 (d, 1H, J = 7.79 Hz, 6′-H), 7.30 (s, 1H, 2′-H), 7.62 (m, 2H, 6-H, 2-H), 7.87 (m, 1H, α-H), 7.94 (m, 1H, β-H). ^13^C-NMR (100 MHz, acetone-d_6_) δ (ppm) 28.48 (C-7′’& C-8′’), 78.20 (C-6′’), 115.67 (C-2), 116.34 (C- 5), 116.86 (C-5′), 119.66 (C-α), 121.71 (C-3′), 122.43 (C-4′), 122.97 (C-6), 127.93 (C-2′), 128.43 (C-6′), 130.96 (C-1′), 132.16 (C-5′’), 132.51 (C-1), 144.53 (C-β), 146.30 (C-4), 146.30 (C-3), 157.87 (C-4′), 187.90 (C = O). ESI-HRMS *m/z* calcd. for C_20_H_18_O_4_ [M + H]^+^ 323.1278 found 323.1285.

#### (E)-3–(2,3-dihydroxyphenyl)-1–(2,2-dimethyl-2H-chromen-6-yl)prop-2-en-1-one (18)

1–(7-methoxy-2,2-dimethyl-2H-chromen-6-yl)ethan-1-one; 2,3-bis(methoxymethoxy)benzaldehyde, yield: m = 320 mg (60%), TLC Rƒ 0.56 (n-hexane/ethyl acetate 2/1), ^1^H-NMR (400 MHz, acetone-d_6_) δ (ppm) 1.43 (s, 6H, 7′’-H & 8′’-H), 5.80 (d, 1H, J = 10.26 Hz, 5′’-H), 6.50 (d, 1H, J = 10.26 Hz, 4′’-H), 6.71 (t, 1H, J = 7.92 Hz, 5-H), 6.82 (d, 1H, J = 8.30 Hz, 5′-H), 6.90 (d, 1H, J = 7.82 Hz, 4-H), 7.28 (d, 1H, J = 7.82 Hz, 6-H), 7.81 (m, 2H, α-H, 2′-H), 7.92 (d, 1H, J = 8.30 Hz, 6′-H), 8.12 (d, 1H, J = 15.63 Hz, β-H). ^13^C-NMR (100 MHz, acetone-d_6_) δ (ppm) 28.28 (C-7′’ & C-8′’), 78.03 (C-6′’), 116.69 (C-5′), 117.16 (C- 4), 120.12 (C-5), 120.15 (C-6), 121.55 (C-1), 122.19 (C-4′’), 122.21 (C-α), 123.05 (C-3′), 127.75 (C-2′), 130.78 (C-6′), 132.00 (C-5′’), 132.29 (C-1′), 139.23 (C-β), 145.75 (C-3), 146.37 (C-2), 157.73 (C-4′), 188.14 (C = O). ESI-HRMS *m/z* calcd. for C_20_H_18_O_4_ [M + H]^+^ 323.1278 found 323.1271.

#### (E)-3–(2,5-dihydroxyphenyl)-1–(2,2-dimethyl-2H-chromen-6-yl)prop-2-en-1-one (19)

1–(7-methoxy-2,2-dimethyl-2H-chromen-6-yl)ethan-1-one; 2,5-bis(methoxymethoxy)benzaldehyde, yield: m = 150 mg (57%), TLC Rƒ 0.51 (n-hexane/ethyl acetate 3/1) ^1^H-NMR (400 MHz, acetone-d_6_) δ (ppm) 1.44 (s, 6H, 7′’-H & 8′’-H), 5.82 (d, 1H, J = 9.77 Hz, 5′’-H), 6.52 (d, 1H, J = 9.77 Hz, 4′’-H), 6.82 (m, 3H, 3-H & 4-H & 5′-H), 7.22 (s, 1H, 6-H), 7.75 (d, 1H, J = 15.63 Hz, α-H), 7.86 (s, 1H, 2′-H), 7.93 (d, 1H, J = 8.30 Hz, 6′-H), 8.09 (d, 1H, J = 15.63 Hz, β-H). ^13^C-NMR (100 MHz, acetone-d_6_) δ (ppm) 28.28 (C-7′’& C-8′’), 78.01 (C-6′’), 114.37 (C-6), 116.69 (C- 3), 117.60 (C-4), 119.63 (C-1), 121.53 (C-α), 121.85 (C-5′), 122.21 (C-4′’), 123.27 (C-3′), 127.74 (C-2′), 130.76 (C-1′), 131.98 (C-6′), 132.30 (C-5′’), 139.34 (C-β), 150.90 (C-2), 151.15 (C-5), 157.70 (C-4′), 188.13 (C = O). ESI-HRMS *m/z* calcd. for C_20_H_18_O_4_ [M + H]^+^ 323.1278 found 323.1273.

#### (E)-3–(3,5-dihydroxyphenyl)-1–(2,2-dimethyl-2H-chromen-6-yl)prop-2-en-1-one (20)

1–(7-methoxy-2,2-dimethyl-2H-chromen-6-yl)ethan-1-one; 3,5-bis(methoxymethoxy)benzaldehyde, yield: m = 125 mg (77%), TLC Rƒ 0.40 (n-hexane/ethyl acetate 3/1) ^1^H-NMR (400 MHz, acetone-d_6_) δ (ppm) 1.44 (s, 6H, 7′’-H & 8′’-H), 5.82 (d, 1H, J = 10.26 Hz, 5′’-H), 6.45 (s, 1H, 4-H), 6.52 (d, 1H, J = 10.26 Hz, 4′’-H), 6.75 (s, 2H, 2-H & 6-H), 6.84 (d, 1H, J = 8.30 Hz, 5′- H), 7.56 (d, 1H, J = 14.16 Hz, α-H), 7.68 (d, 1H, J = 14.16 Hz, β-H), 7.88 (s, 1H, 2′-H), 7.95 (d, 1H, J = 8.30 Hz, 6′-H), 8.43 (s, 2H, 2x OH). ^13^C-NMR (100 MHz, acetone-d_6_) δ (ppm) 28.50 (C-7′’& C-8′’), 78.28 (C-6′’), 105.63 (C-4), 107.80 (C- 2 & C-6), 116.95 (C-5′), 121.76 (C-α), 122.36 (C-3′), 122.60 (C-4′’), 128.06 (C-5′’), 131.08 (C- 1′), 132.21 (C-6′), 138.11 (C-1), 144.27 (C-β), 158.10 (C-4′), 159.73 (C-3 & C-5), 188.00 (C = O). ESI-HRMS *m/z* calcd. for C_20_H_18_O_4_ [M + H]^+^ 323.1278 found 323.1279.

### Inhibition assay of luciferase

25 µL of luciferase protein (c = 0.6 µg/mL, Sigma-Aldrich, recombinant expressed in E.coli) in Trisbuffer (10 mM Tris, 10 mM KCl, 1 mM EGTA, 30% glycerol) pH7.4 were added to each well of a white 96 well plate (Brand). The compounds were added in 1 µL DMSO at various concentrations. After incubation of the enzyme with the compounds for 10 min at room temperature, 80 µL of FLuc buffer (25 mM glycylglycin, 4 mM EGTA, 15 mM MgSO_4_, 15 mM PPB) was added and the light signal was immediately measured using a multiplate reader (Clariostar, BMG) with a gain of 2500 and a measurement interval time of 2 s. Inhibition was calculated in relation to the control, enzyme treated with vehicle (DMSO).

### Molecular docking

Molecular modelling was performed using AutoDock Vina ((http://vina.scripps.edu/).^21^ The crystal structure of firefly luciferase in complex with PCT124 (ataluren)-AMP (PDB ID: 3IES, 2.00 Å resolution) were edited in Chimera 1.14.^22^ This includes manually removing all H_2_O molecules and the ataluren molecule from the active site, adding hydrogens and Gasteiger charge. Ligands were prepared using ChemDraw 17.0 and Chem3D 17.0 (http://www.cambridgesoft.com) to perform MM2 energy minimisation. The scoring grid (20.7 × 25.3 × 33.0) was centered to x-center: −30.743, y-center: 7.13472, and z-center-14.3621. For result visualisation and figure preparation UCSF Chimera and Discovery Studio 2021 (www.3ds.com) was used to show interactions and H-bonding of the highest affinity binding mode (kcal/mol).

### Statistics

OriginPro 2021b (64-bit) SR2 (9.8.5.212) (OriginLab) was used to determine the inhibition and IC_50_ values and prepare the figures. The IC_50_ values were calculated from the sigmoidal fit of the log scale concentration without fixing the parameters.

## Results and discussion

In contrast to xanthohumol C (**1**) (c = 10 µM), the initially investigated hop compound, the simplified derivative (**2**) (c = 10 µM), which lacks additional substituents at the A-ring, demonstrated an inhibition of FLuc by 30% (Figure 1).

**Figure 2.**
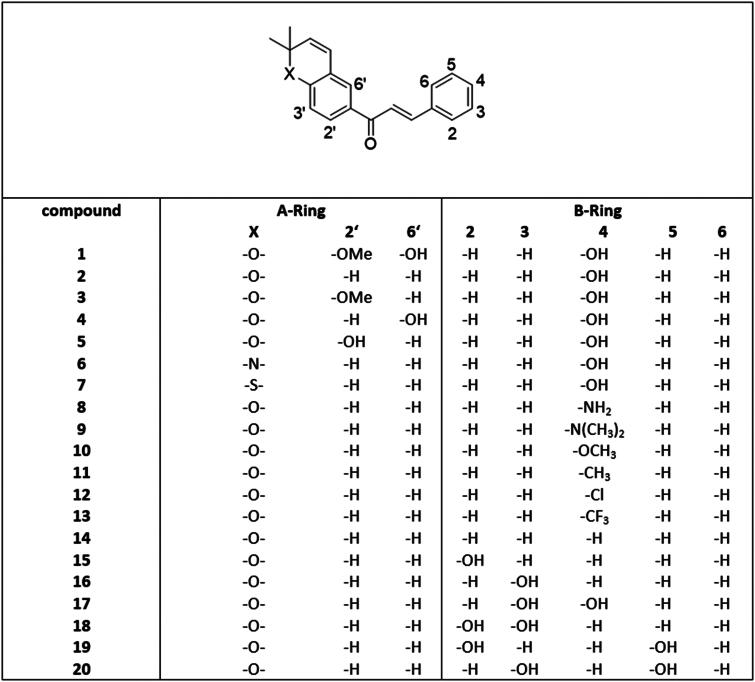
Structures of pyranochalcones with different substitution pattern on A- and B-ring.

Accordingly, the objective was to examine the impact of the A- and B-ring substitution pattern of chalcones on FLuc inhibition, in order to ultimately assess the applicability of reporter gene assays for this class of compounds. To this end, further 18 distinct chalcones, exhibiting varying substitution patterns on both the A- and B-rings, were synthesised (Figure 2) and subjected to investigation for the potential to inhibit FLuc.

### Synthesis

**Scheme 1.**
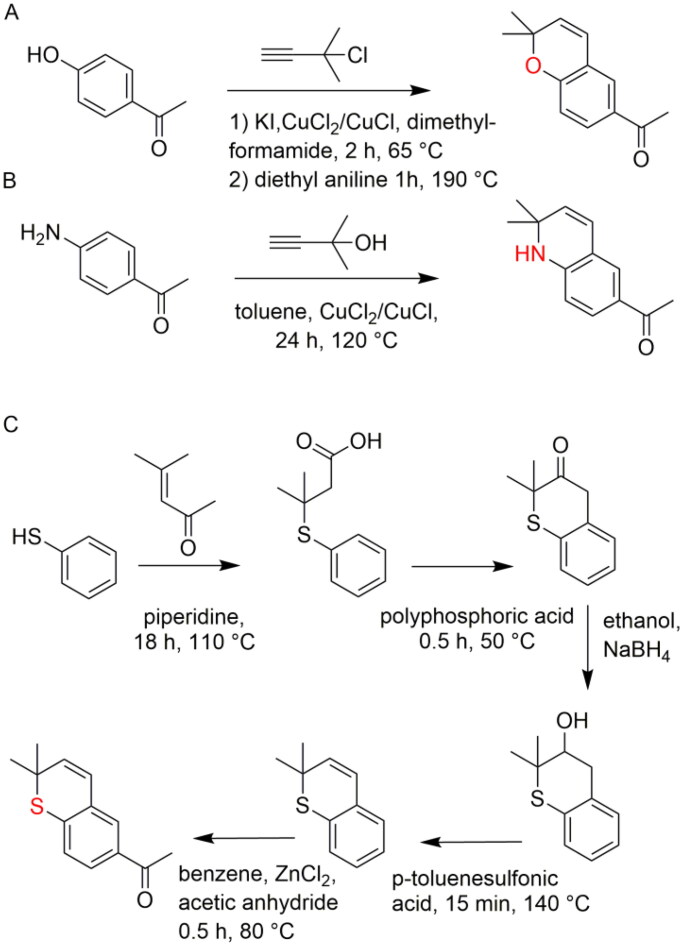
Synthesis route of A-rings with different heteroatoms.

The A-rings of the derivatives with oxygen as heteroatom (compounds **3**, **4** and **5**) were obtained via multistep synthetic routes consisting of protection of hydroxyl groups as methoxymethyl(MOM)ether, methylation if necessary, addition of a prenyl group, rearrangement of the prenyl group, deprotection and ring closure to a chromane-like ring. The A-rings of compounds **2** and **6** were synthesised (Scheme 1A and B) according to the methods described by *Bohlmann* et al. and *Hamann* et al. starting with the condensation of butyne and 4-hydroxyacetophenone or 4-aminoacetophenone.^23^^,^^24^ The preparation of the thiochromane derivative **7** was somewhat more complex, since the reaction analogous to the chromane-like ring of 4-thioacetophenone with 3-chloro-3-methyl-1-butyne did not lead to the desired product. Accordingly, an alternative synthetic route (Scheme 1C) was selected, starting with thiophenol. Following the addition of dimethylacrylic acid to thiophenol, the ring was closed with phosphoric acid, after which the carbonyl group was reduced with sodium borohydride.^25^ After the elimination of water by the addition of *p*-toluenesulfonic acid and the addition of an acetyl group, the desired A-ring with sulphur as a heteroatom was obtained and identified by 1D and 2D NMR. The acetylation product (Scheme 1B) was identified by HMBC, which revealed an interaction between H-2′ with H-4′’ and H-5′’, and the multiplicity observed in the 1D NMR data supports the 2D results. All A-rings were then condensed with the appropriate (protected) aldehyde, thereby yielding the final products.^5^^,^^6^

The inhibitory activity of all chalcones was evaluated at a concentration of 10 µM on purified recombinant firefly luciferase, as this concentration would also be used in the reporter gene assay to determine neurogenic activity.

**Figure 3.**
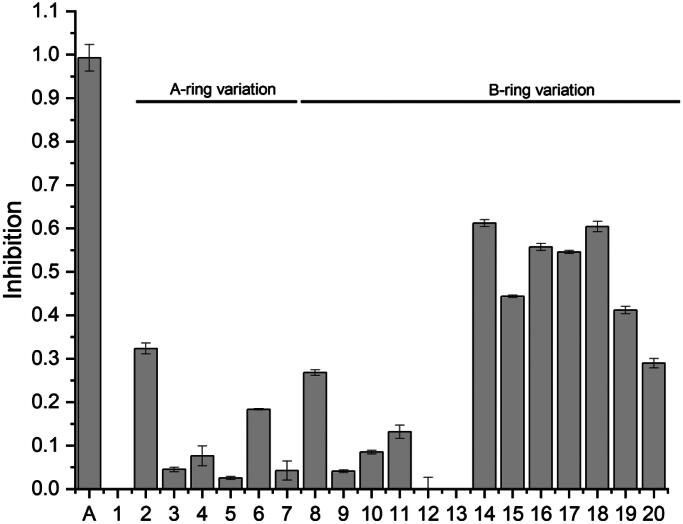
Luciferase inhibition (%) of all chalcones synthesised in a concentration of 10 µM (*n* = 3).

The inhibition of FLuc displays considerable variability, contingent upon the substitution pattern observed in both the A and B-rings (Figure 3).

### Structure-activity relationship

Compound **1,** the neurogenic natural occurring chalcone, showed no inhibition of FLuc at a concentration of 10 µM, in contrast to the simplified structure of compound **2** (Figure 1). The difference between these compounds is attributed to the substitution pattern on the A-ring. To elucidate the influence of the substitution pattern on the A-ring, compounds (**3**, **4,** and **5**) were synthesised with varying substitution patterns at the A-ring. Compound **3**, substituted with a methoxy group on position 2′ and compound **5** which has a hydroxyl group on position 2′, both show only slight inhibition, below 5%, at a concentration of 10 µM. The substitution of a hydroxyl group on position 6′ (compound **4**) resulted in a slightly higher inhibition of FLuc, however, below 10% (Figure 3).

Compound **2,** which lacks additional functional groups on the A-ring, demonstrated the most pronounced inhibitory effect. Therefore, the A-ring, which lacks functional groups and resembles a chromane ring, was selected for subsequent experiments. The substitution of oxygen in the chromane-like ring by nitrogen (compound **6**) resulted in a noteable reduction in FLuc inhibition from 32% to 18% (Figure 3). Furthermore, the substitution of oxygen with sulphur (compound **7**) resulted in an inhibitory effect that was less than 10% (Figure 3). Accordingly, oxygen was retained as the heteroatom in the chromane-like ring, and the substitution pattern of the B-ring was altered. Initially, the hydroxyl group at position 4 of compound **2** was substituted with different functional groups. The introduction of an amino group (compound **8**) resulted in an inhibitory effect that was comparable to that of the hydroxyl group (Figure 3). This effect may be attributed to the analogous behaviour of the amino group in hydrogen bonding to a target as the hydroxyl group. This is also supported by the observation that the introduction of a dimethylamine group (compound **9**) results in a reduction in inhibitory activity, which is likely due to the prevention of hydrogen bonding. A reduction in the inhibitory potential is also observed when comparing compound **2** and compound **10**, which differ in that one contains a methoxy group and the other a hydroxyl group at position 4 of the B-ring. The presence of a methyl group at position 4 of the B-ring in compound **11** resulted in an inhibitory effect that is slightly more pronounced than that observed for compound **10** (Figure 3). Moreover, the introduction of electron-withdrawing groups at position 4 (compounds **12** and **13**) has been observed to result in the near-complete elimination of the inhibitory effect. In contrast, a study by Zhang *et al.* demonstrated, that these groups on position 4′ of the A-ring of chalcones lacking the chromane-like ring can facilitate the luciferase inhibition.^18^ The most pronounced inhibitory effect is attributable to a hydrogen atom at position 4 of the B-ring (compound **14**) (Figure 3). Consequently, the inhibitory effect is influenced not only by the functional groups but also by substitution pattern on the B-ring, and this influence was investigated as follows. The substitution of the B-ring with a hydroxyl group at either position 2 (compound **15**) or position 3 (compound **16**) resulted in a notable increase in the inhibitory effect, with the latter exhibiting a more pronounced impact (Figure 3). The substitution of the B-ring with two hydroxyl groups also demonstrated inhibitory potential, contingent on the position. The highest level of inhibitory activity was observed for two hydroxyl groups in the meta position, as exemplified by in compounds **17** and **18** in comparison to compounds **19** and **20** (Figure 3).

Subsequently, compounds exhibiting an inhibition more than 10% at a concentration of 10 µM were subjected to further evaluation concerning FLuc inhibition, including the determination of IC_50._ To obtain the IC_50_ values and construct a concentration-response curve, chalcones were measured in the assay using a series of decreasing concentrations, ranging from 100 µM to 13 nM and from 300 µM to 0.41 nM. Ataluren (A) (c = 0.11 µM), a known luciferase inhibitor, was included as a positive control and the IC_50_ of 0.11 µM is in good agreement with the results of other studies.^12^

**Figure 4.**
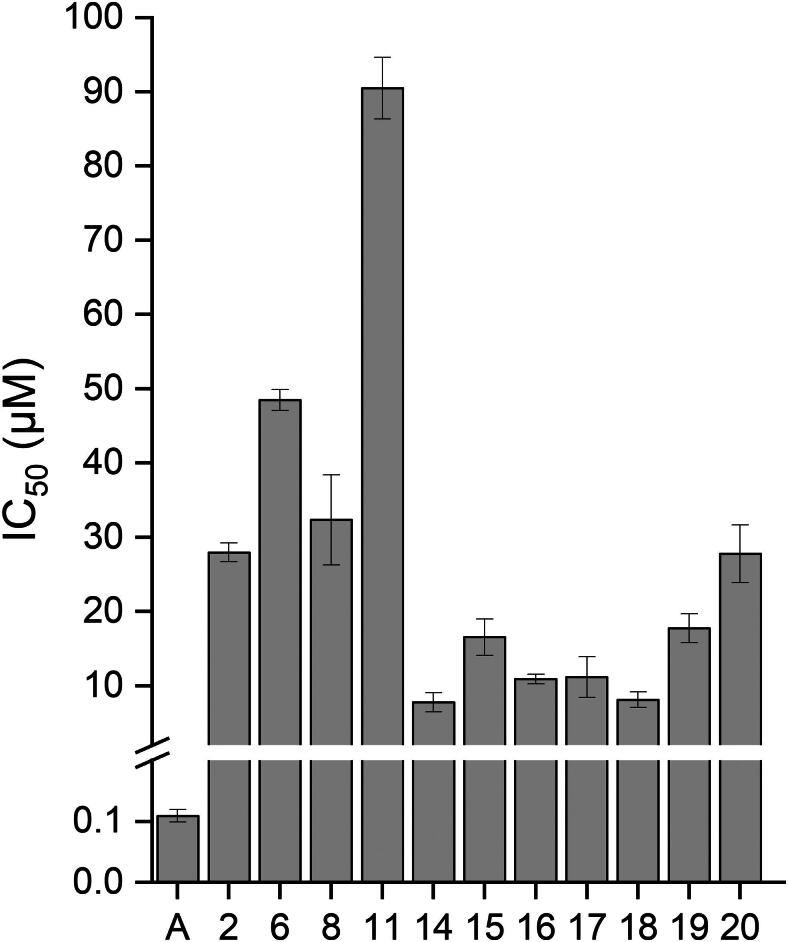
IC_50_ values of compounds showing more than 10% inhibition at a concentration of 10 µM (mean ± standard deviation; *n* = 3).

As illustrated in Figure 4, the IC_50_ of chalcones on FLuc varies with the substitution pattern between concentrations of 7.82 µM and 92.99 µM (Figure 4). Chalcones that lack the chromane-like ring and are substituted on position 4 of the A-ring and position 4 and 5 on B-ring exhibit IC_50_ values ranging from 0.20 to 21.56 µM, which is also dependent on the functional groups.^18^ In this study, the presence of a ­carboxyl group on the B-ring was found to be essential for the observed strong inhibitory effect.^18^

The lowest IC_50_ was determined for compound **14,** ­followed by compound **18**, both with IC_50_s below 10 µM (Figure 4). Other natural products, such as resveratrol and other ­chalcones, with a carboxylic group at the B-ring exhibit IC_50_ values of 2.33 µM and 0.2 µM, respectively 4.94 µM.^11^^,^^18^

### Molecular docking

Moreover, a molecular docking study was performed to explore possible correlations between the *in vitro* results and binding affinities and conformations. Autodock Vina^21^^,^^22^^,^^26^ was used to dock compounds **1**, **2**, **14** and **18** into the X-ray structures of FLuc containing PTC124 (ataluren)-AMP (PDB: 3IES), given that an activation response in Fluc cell-based reporter gene assays for ataluren, a multisubstrate adduct inhibitor, was observed indicating enzyme stabilisation.^12^ Additionally, *in silico* analyses revealed that chalcones lacking the chromane-like ring exhibited a binding pattern to firefly luciferase (FLuc) similar to that observed with PTC124 (ataluren).^18^ The protein was prepared using UCSF Chimera including the addition of hydrogen atoms and the stripping of water. The structures of the ligands were minimised with regard to energy using ChemDraw3D. The size and position of the docking grid were adjusted to include the AMP sub-pocket, which facilitates the formation of a luciferyl adenylate intermediate and the subsequent generation of AMP, oxyluciferin, and light in the bioluminescence reaction.^12^

**Figure 5.**
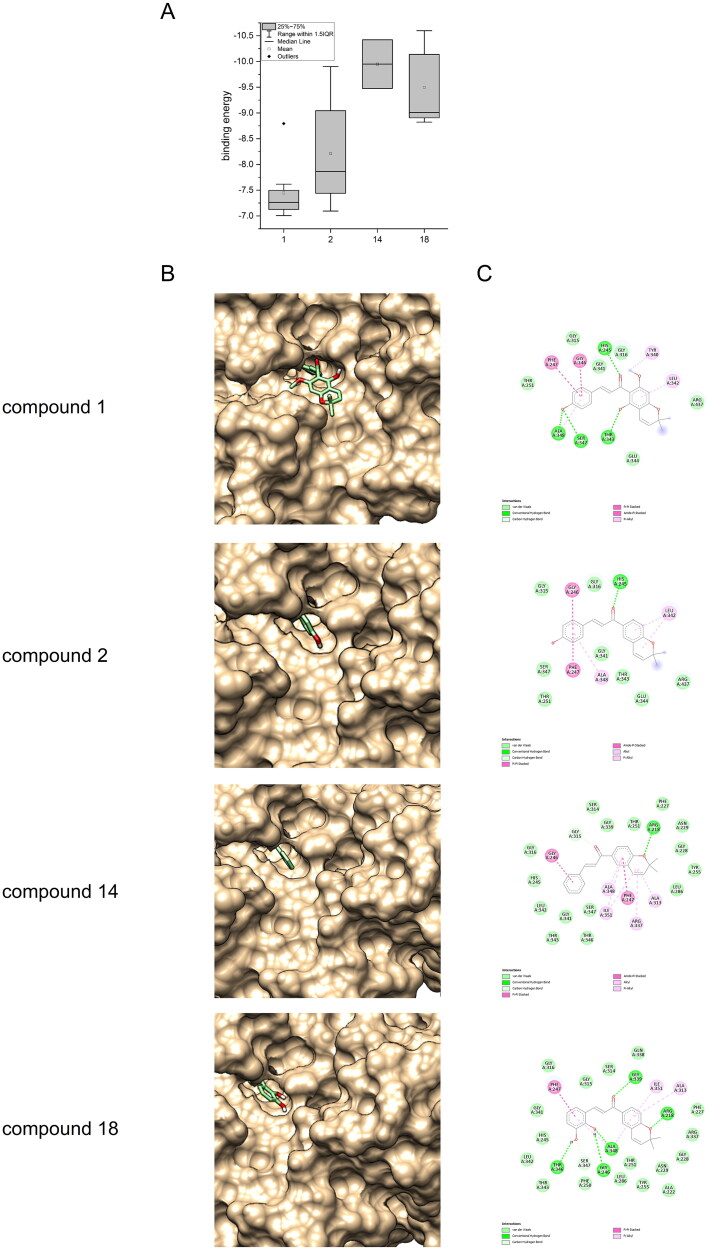
A: binding energies (kcal/mol) of compounds 1, 2, 14, and 18; B: compounds 1, 2, 14, and 18 shown as green sticks at the target site; C: interactions between compounds 1, 2, 14, and 18, and the key amino acid residues within the targeted site. The compound structures are shown as gray structure formula while the amino acid residues are shown as green and pink balls.

Figure 5A illustrates the disparate binding energies exhibited by FLuc for the different ligands. For compound **1,** the range of binding energies was observed to be from −7.007 to −8.794 kcal/mol, with the latest value observed at the sole position within the active binding site. The binding energies of compounds **2**, **14** and **18** ranged from −9.902 to −7.699 kcal/mol, −10.422 to −9.475 kcal/mol and −10.598 to −8.823 kcal/mol.

The results of the docking analysis demonstrate that a good fit for compound **1** within the FLuc binding pocket (Figure 5B) is achieved by entering the B-ring, with a deeper engagement within the active site hindered by the methoxy and hydroxyl groups present on the A-ring. These findings are consistent with those observed in the docking analysis of compound **2**, which also exhibits a similar orientation within the FLuc binding pocket (Figure 5B), albeit with a deeper penetration into the active site. In contrast to compound **1**, compound **2** demonstrates an interaction with ALA348 near the hydrophobic pocket, with the B-ring. This indicates that an unsubstituted A-ring provides a superior fit within the pocket. This findings align with the observations made in the study of Zhang et al., which demonstrated that chalcones with a simple substitution pattern (i.e., only position 4′) and lacking the chromane-like ring also enter the pocket with the B-ring.^18^ Furthermore, with regards to isoflavonoids, the less polar isoflavonoids with O-methylation on the B-ring are to be preferred over those with a hydroxyl goup.^17^

Furthermore, the docking results indicate that the molecule undergoes a 180° rotation when there is no hydroxyl group present on the B-ring, thereby entering the pocket with the A-ring. In addition, compound **14** demonstrated this orientation, with the lowest binding energy in the docking and exhibited the highest inhibitory activity in the *in vitro* experiments. As illustrated in Figure 5C, compound **14** shows a strong binding interaction, predominantly driven by π-π-stacking and π-alkyl stacking of the B-ring with GLY246 and the A-ring with ALA348, ILE351, PHE247, ARG337, ALA313, and hydrogen bonding with the A-ring and ARG218. The study of Kenda *et al.* also identified two possible orientations for isoflavonoids, differing by a 180° rotation of the bicyclic core scaffold.^17^ The substitution of the B-ring with hydroxyl groups at positions other than position 4 appears to support the binding to FLuc, as evidenced by the high binding energies and the second lowest IC_50_ exhibited by compound **18**, which contains hydroxyl groups in positions 2 and 3 of the B-ring. Based on the results of the docking simulation, this may be attributed to stabilisation by hydrogen bonding between the hydroxyl groups and amino acid residues THR346, GLY246 and ALA348 (Figure 5C). The docking results indicate that all inhibitors interact with ALA348. However, this is not the case for xanthohumol C, which also shows no inhibitory effect *in vitro*. With the exception of xanthohumol C, the pyranochalcones demonstrated a high degree of binding affinity for the D-luciferin binding pocket in the molecular docking. This observation suggests that the luciferase inhibitors may compete with D-luciferin and/or act as multisubstrate adduct inhibitors, similar to ataluren—particularly since the PDB structure selected for molecular docking was derived from the multisubstrate AMP–ataluren complex.^12^ Accordingly, to clarify the binding interactions, advanced studies are required—such as co-crystallization with AMP, as demonstrated in the case of ataluren,^12^ or NMR-based protein–ligand interaction studies.

## Conclusion

Pyranochalcones have been shown to inhibit firefly luciferase (FLuc), with the extent of inhibition dependent on the substitution patterns present on both the A- and B-rings. Even slight modifications in the molecular structure lead to significant changes in inhibition of luciferase activity. Furthermore, *in silico* molecular docking analyses provide potential explanations for the observed selectivity of the compounds towards FLuc, based on the structural characteristics. Given that the IC_50_ values of luciferase inhibition fall within concentration ranges commonly used in both *in vivo* and *in vitro* assays, caution is warranted when interpreting results from reporter gene assays involving FLuc and pyranochalcones. This becomes particularly important when conducting structure–activity relationship (SAR) studies using reporter gene assays, as even minor structural modifications—as demonstrated—can result in significant differences in luciferase inhibition. Furthermore, recognising that the strength of *in vitro* inhibition of FLuc may not directly correlate with the FLuc stabilising effect in cells is essential. Attempting to establish such a correlation would require the isolation of the distinct impacts of the compounds on cellular processes, rather than providing an accurate representation of the overall effects. It cannot be excluded that compounds that inhibit FLuc may also be of interest against the actual target.^10^ Consequently, alternative assays employing readouts other than FLuc are necessary to assess activity of the promising class of pyranochalcones, as was done in the case of xanthohumol C using immunostaining.^5^

## Supplementary Material

20250417_Supplementary Information wo authors.pdf

## Data Availability

The datasets in this study are available from the authors upon reasonable request.
