# Supplementary material for: Chalcones inhibit firefly bioluminescence dependent on A and B-ring substitution pattern – a structure-activity study combined with molecular docking
Source: J Enzyme Inhib Med Chem. 2025 Jun 9;40(1):2509657. doi: 10.1080/14756366.2025.2509657 (PMC12150632; doi:10.1080/14756366.2025.2509657)

Supplementary Information

Chalcones inhibit Firefly Bioluminescence dependent on A and B-Ring substitution pattern – a structure-activity study combined with molecular docking

Inhalt

1H and ^{13C}-NMR of compound 3 2

^{1}H and ^{13}C-NMR of compound 4 3

^{1}H and ^{13}C-NMR of compound 5 4

^{1}H and ^{13}C-NMR of compound 6 5

^{1}H, ^{13}C-NMR and HMBC of compound 7 6

^{1}H and ^{13}C-NMR of compound 8 8

^{1}H and ^{13}C-NMR of compound 9 9

^{1}H and ^{13}C-NMR of compound 10 10

^{1}H and ^{13}C-NMR of compound 11 11

^{1}H and ^{13}C-NMR of compound 13 12

^{1}H and ^{13}C-NMR of compound 14 13

^{1}H and ^{13}C-NMR of compound 15 14

^{1}H and ^{13}C-NMR of compound 16 15

^{1}H and ^{13}C-NMR of compound 17 16

^{1}H and ^{13}C-NMR of compound 18 17

^{1}H and ^{13}C-NMR of compound 19 18

^{1}H and ^{13}C-NMR of compound 20 19

1H and 13C-NMR of compound 3

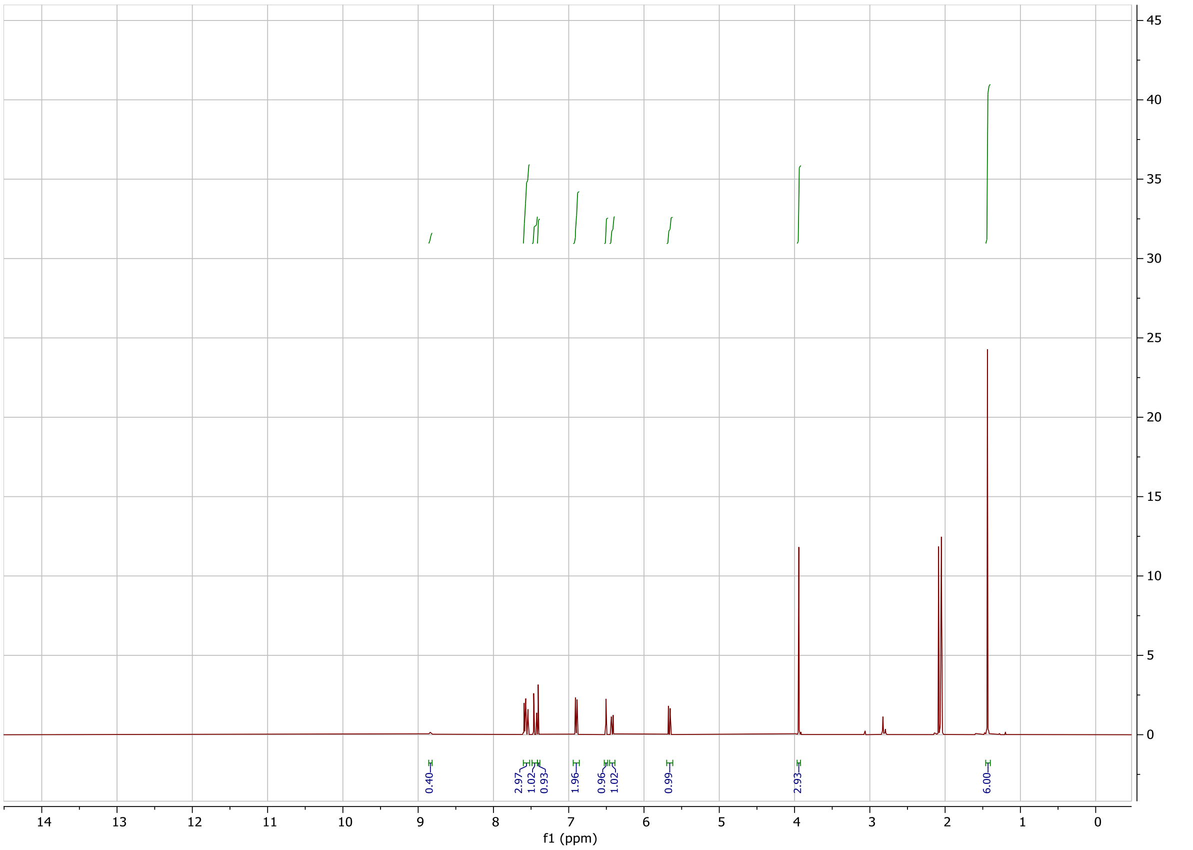

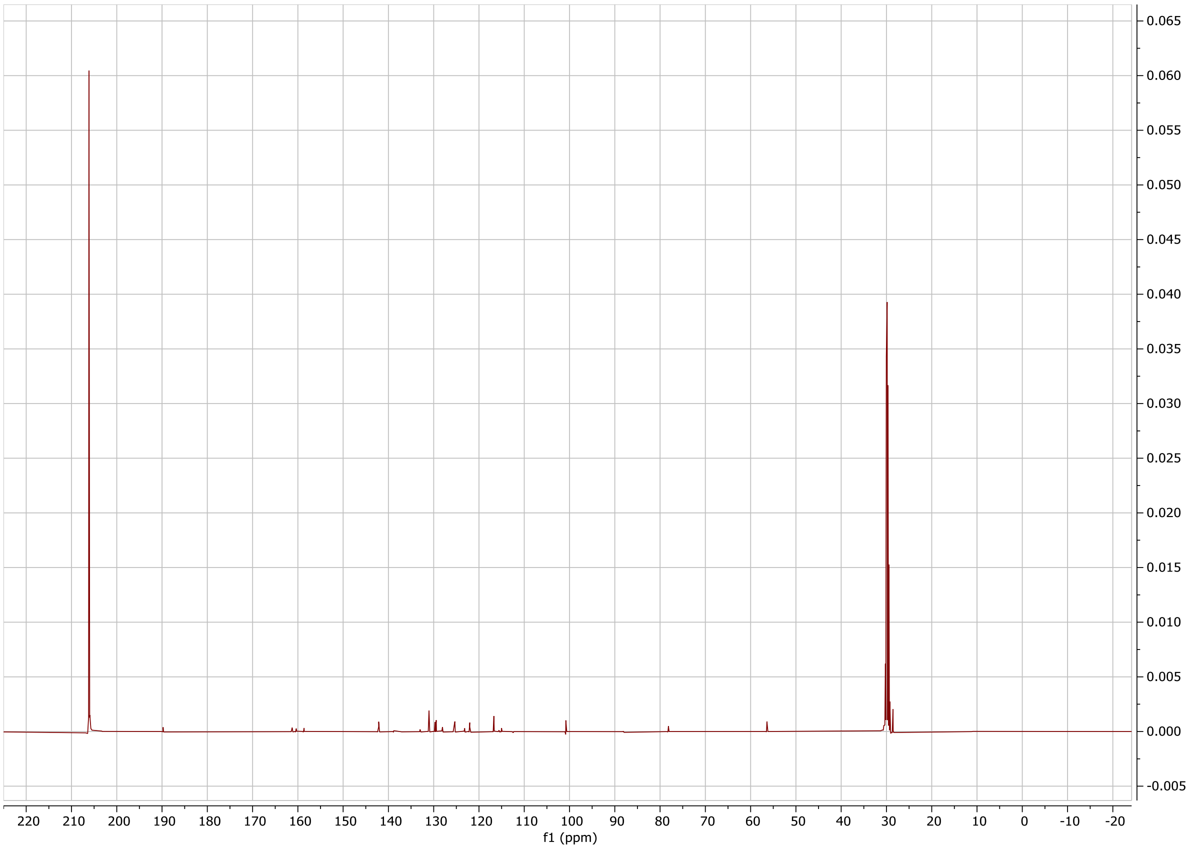

^{1}H and ^{13}C-NMR of compound 4

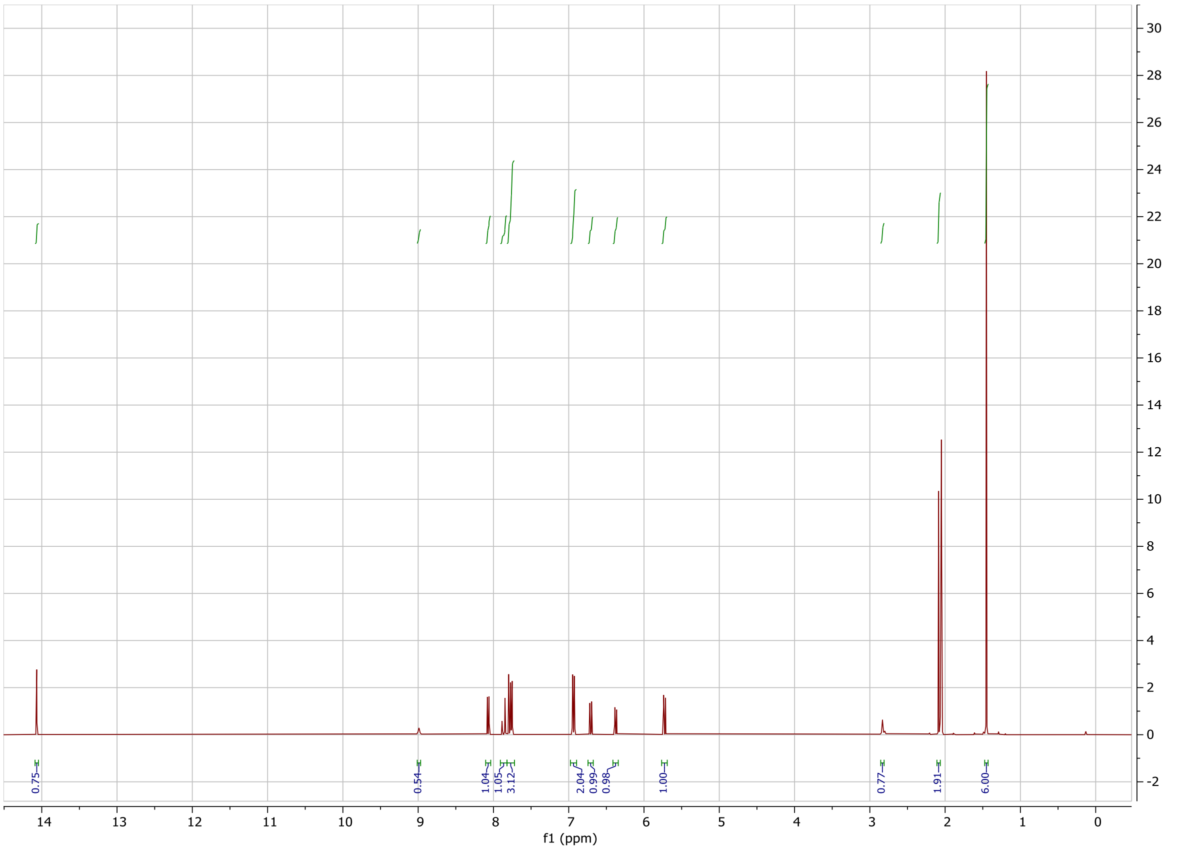

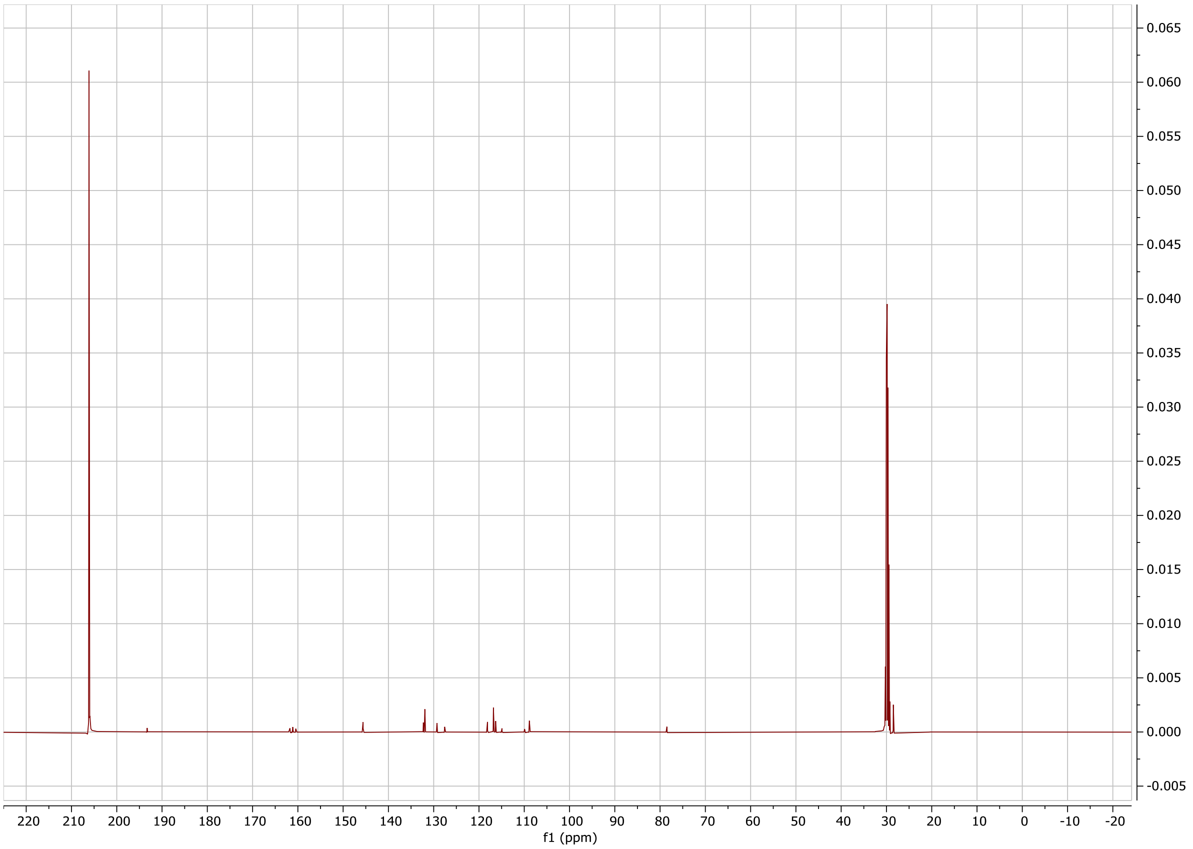

^{1}H and ^{13}C-NMR of compound 5

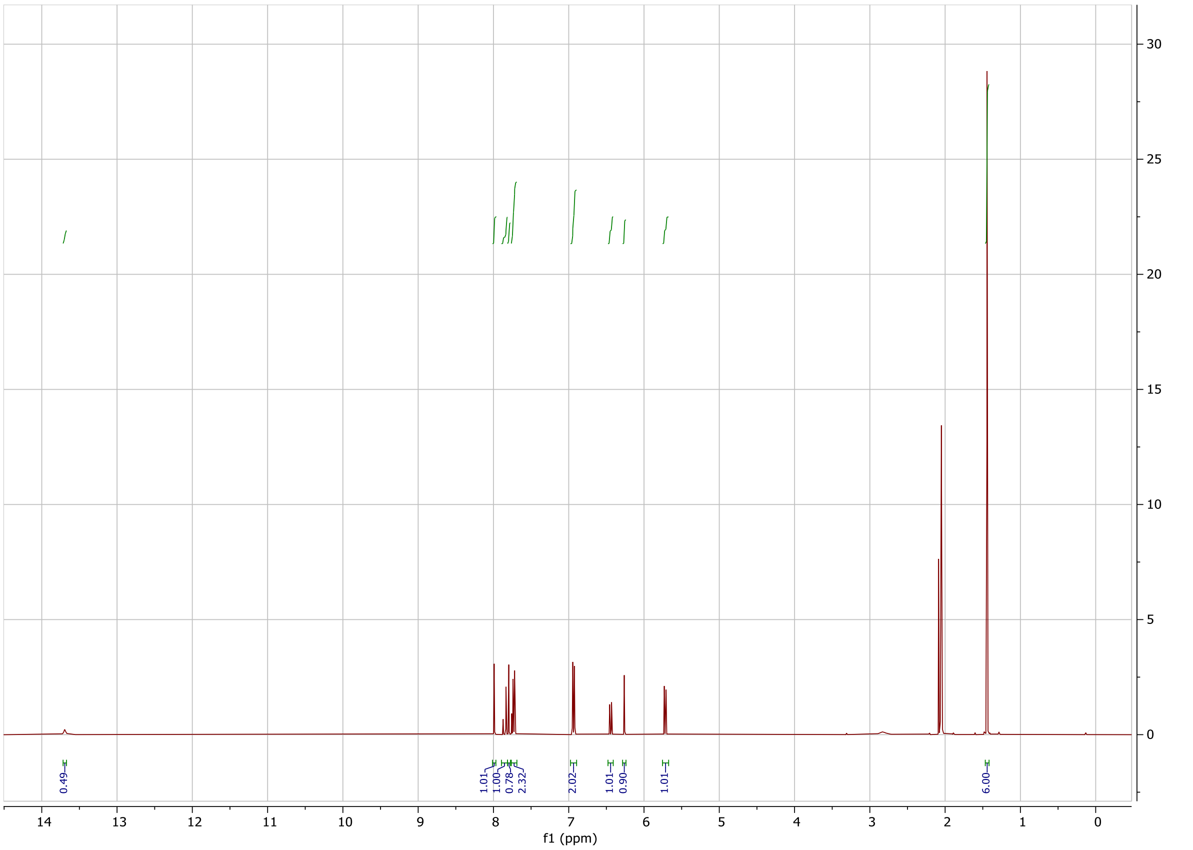

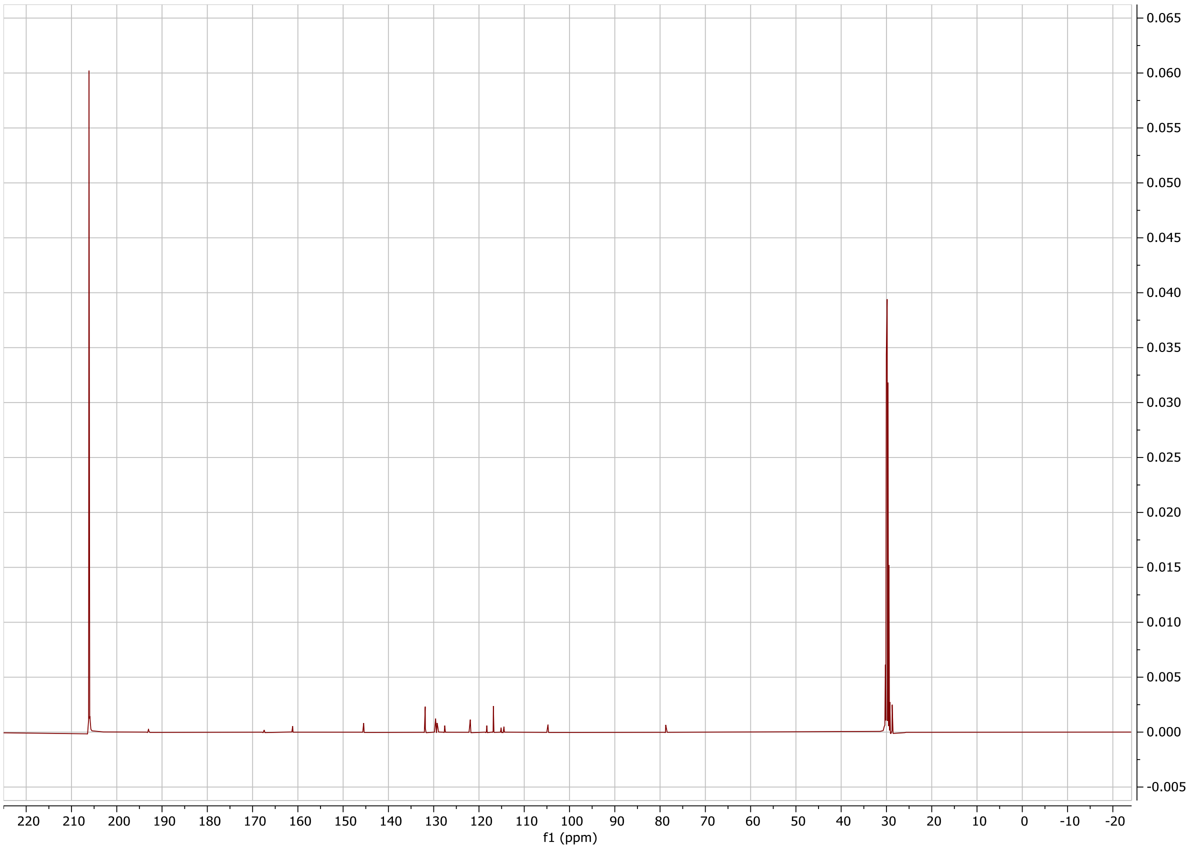

^1H and ^{13}C-NMR of compound 6

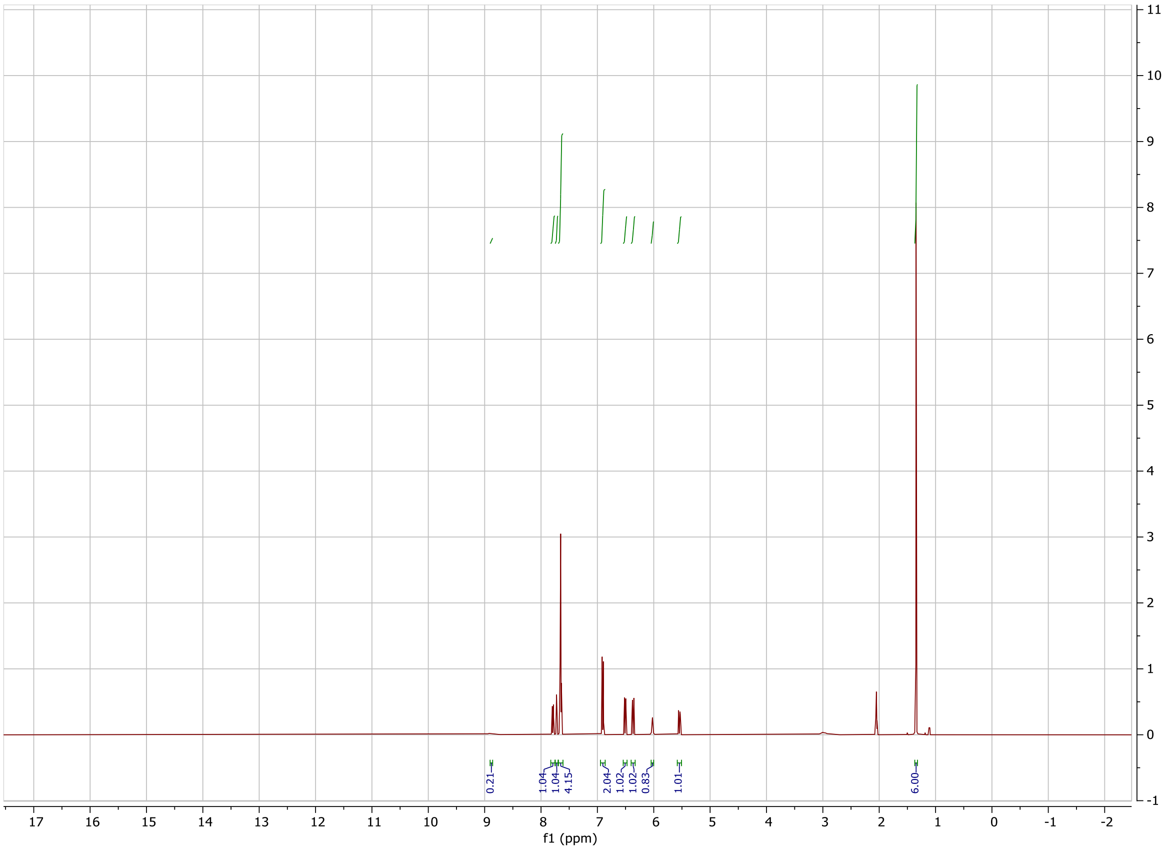

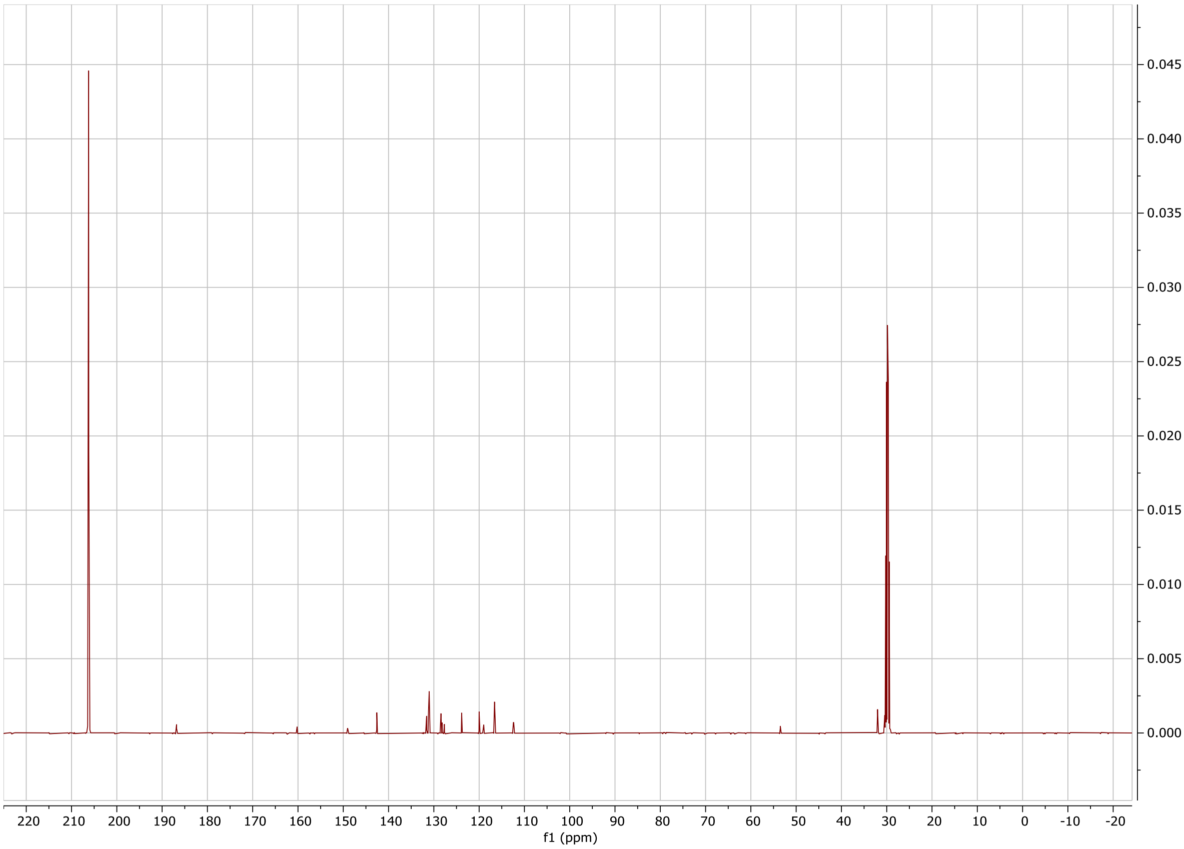

^{1}H, ^{13}C-NMR and HMBC of compound 7

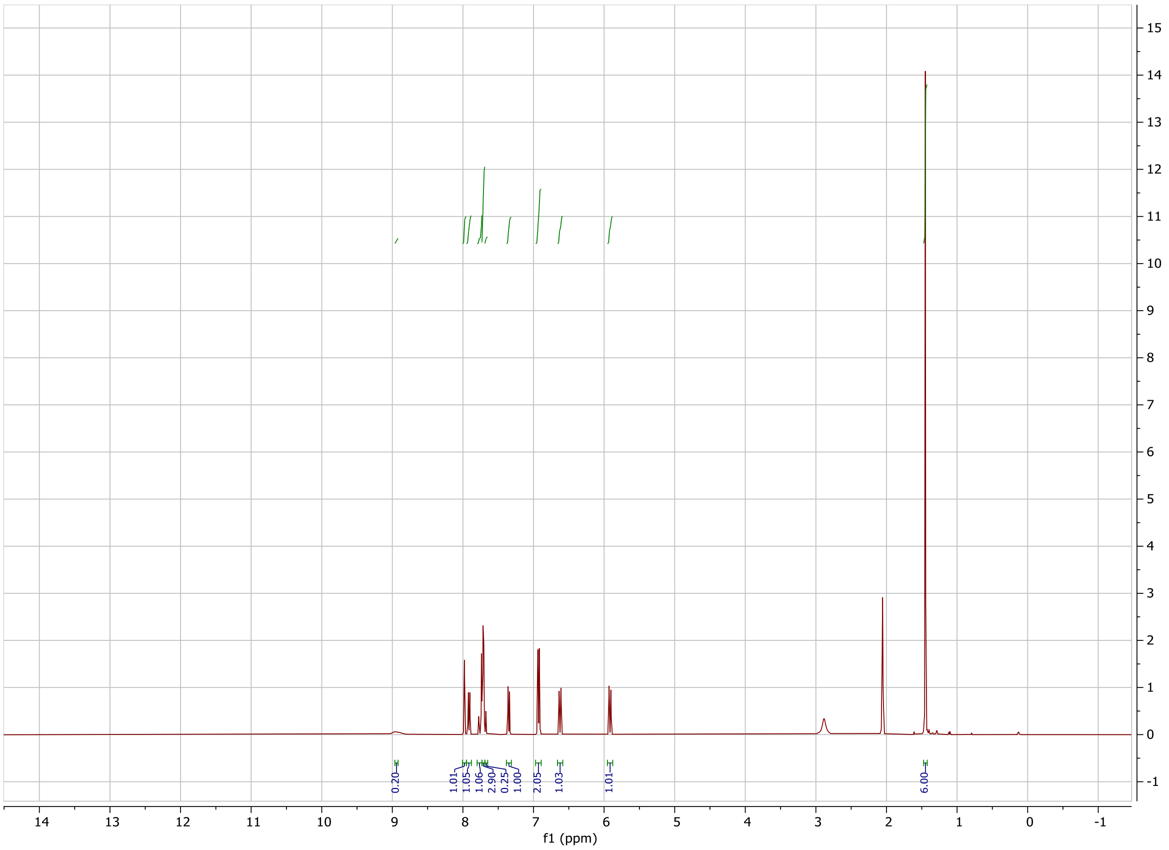

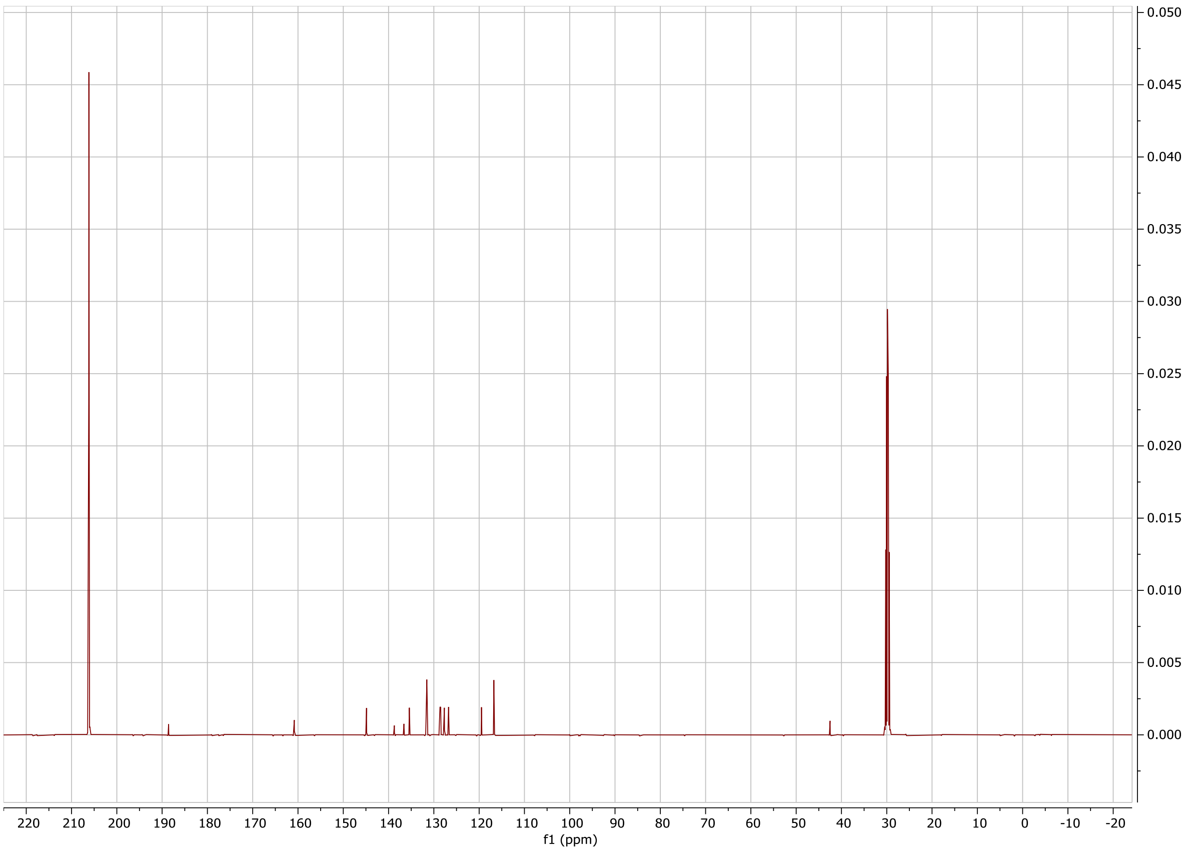

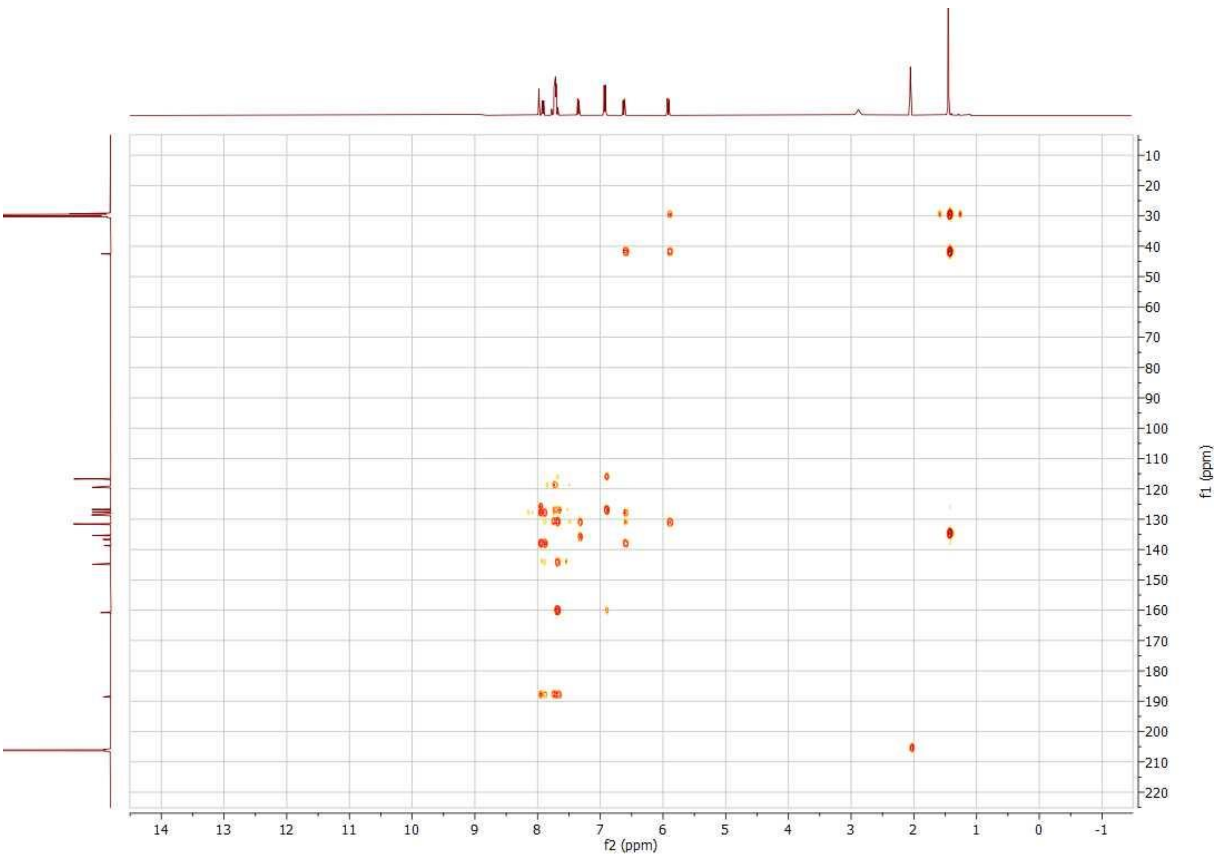

f1 (ppm)
f2 (ppm)

^{1}H and ^{13}C-NMR of compound 8

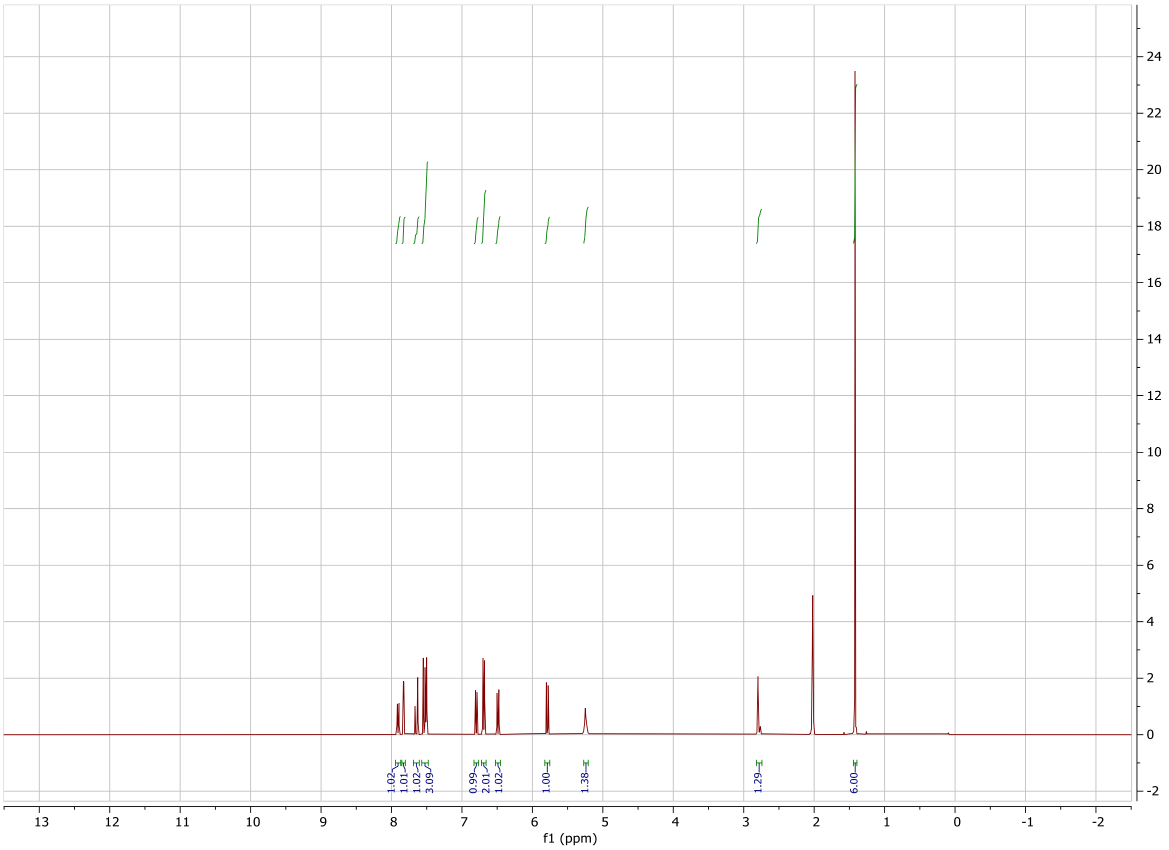

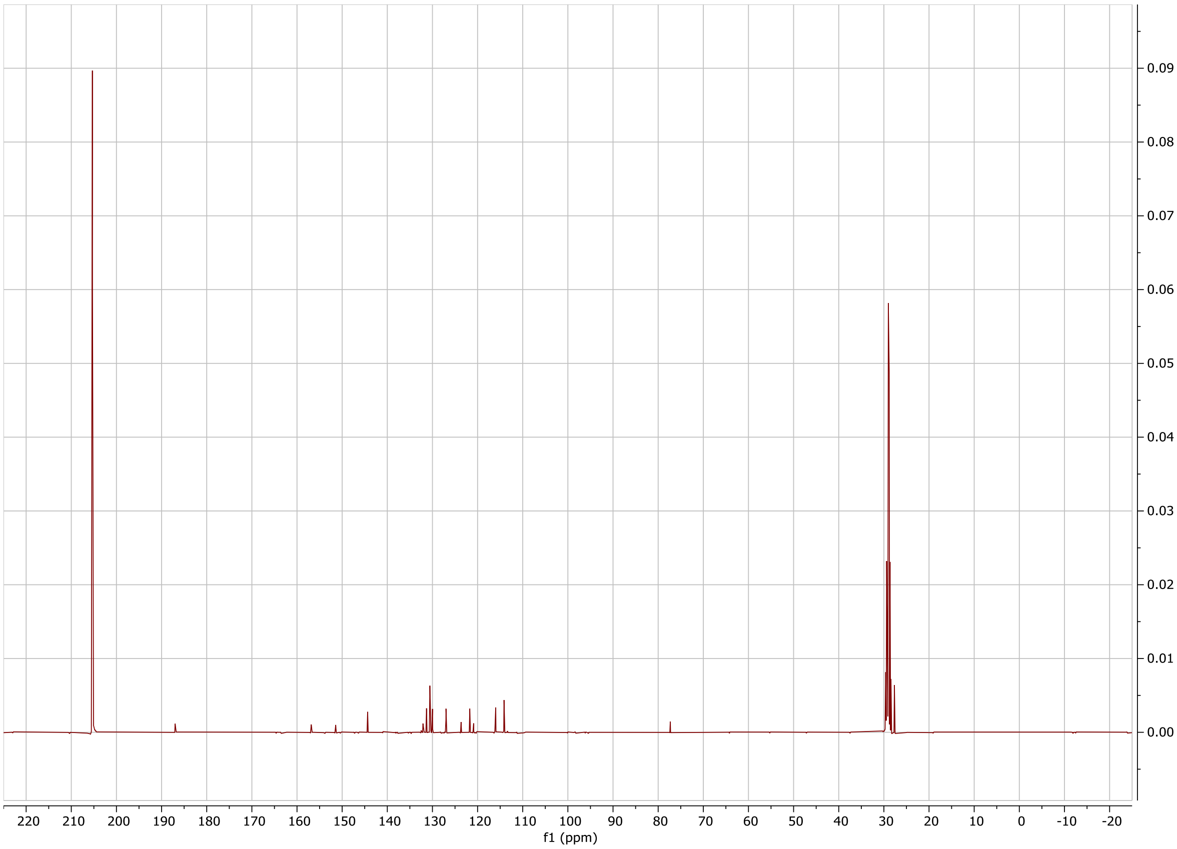

^{1}H and ^{13}C-NMR of compound 9

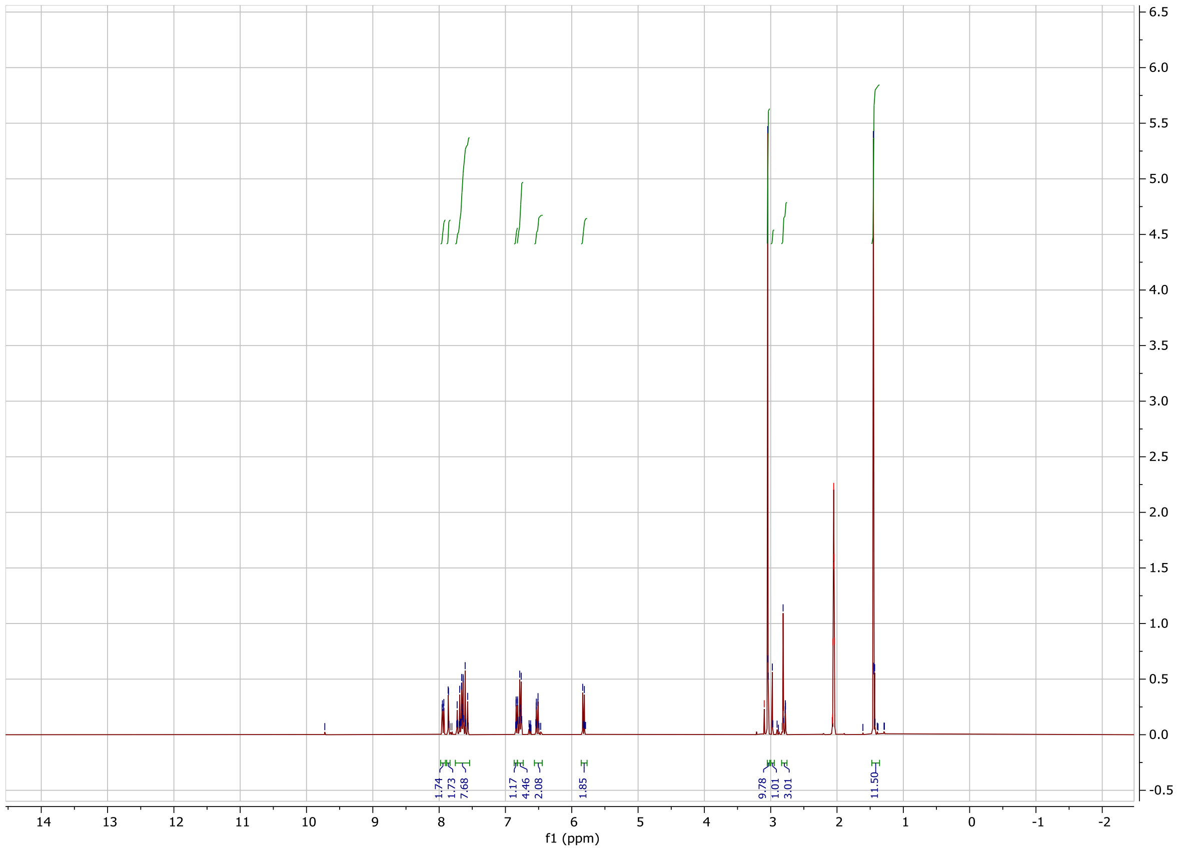

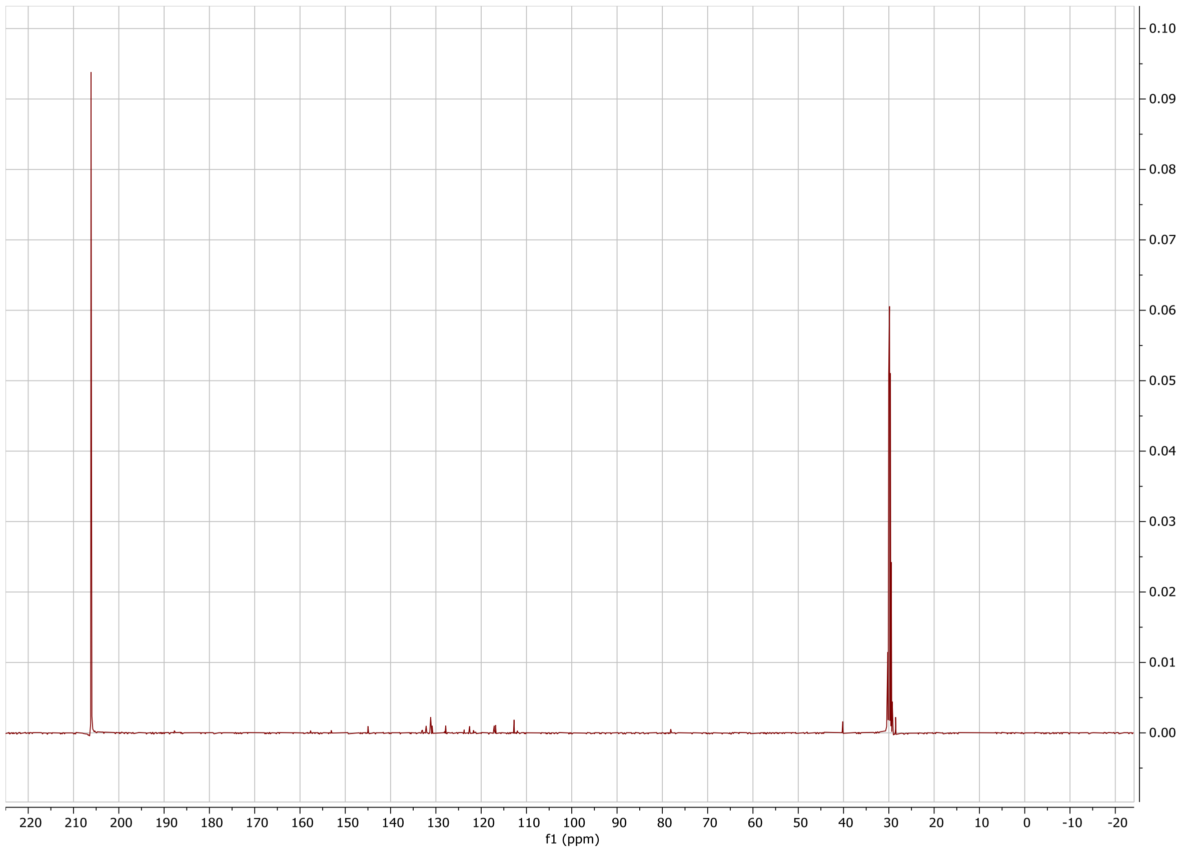

^1H and ^{13}C-NMR of compound 10

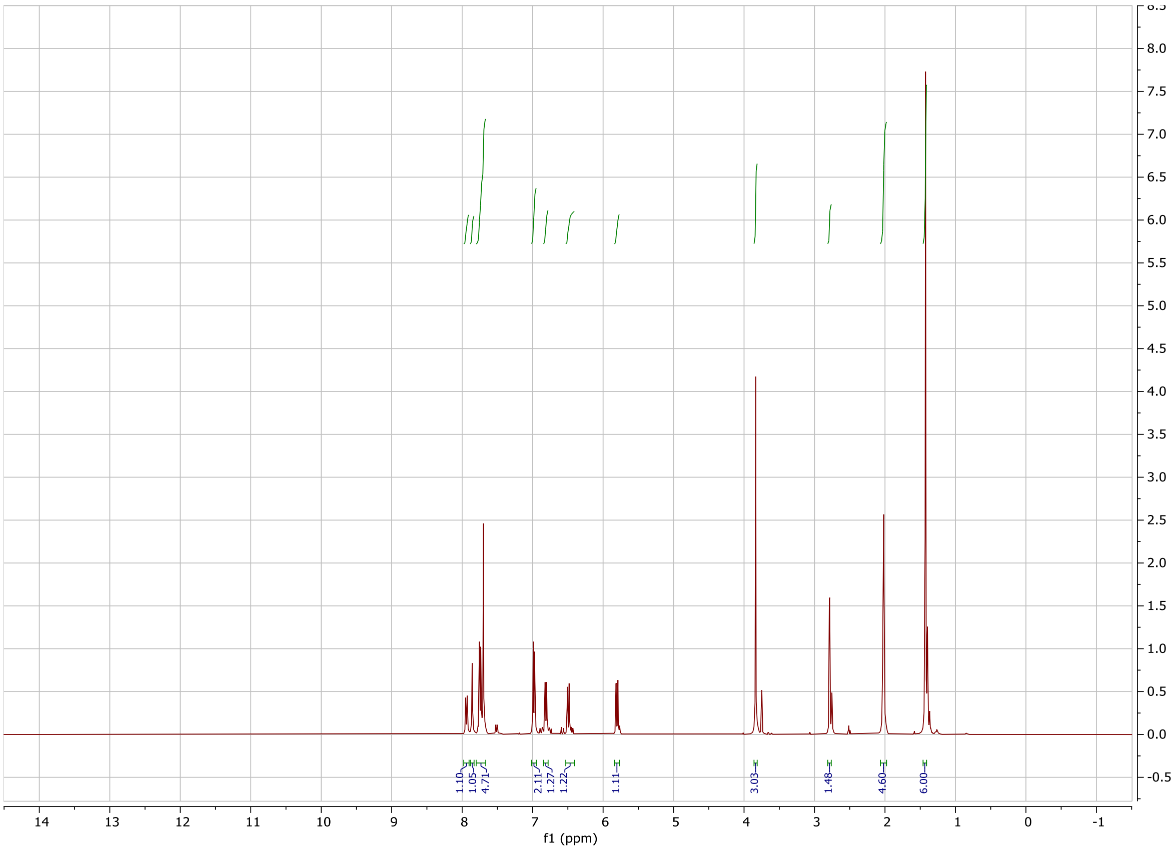

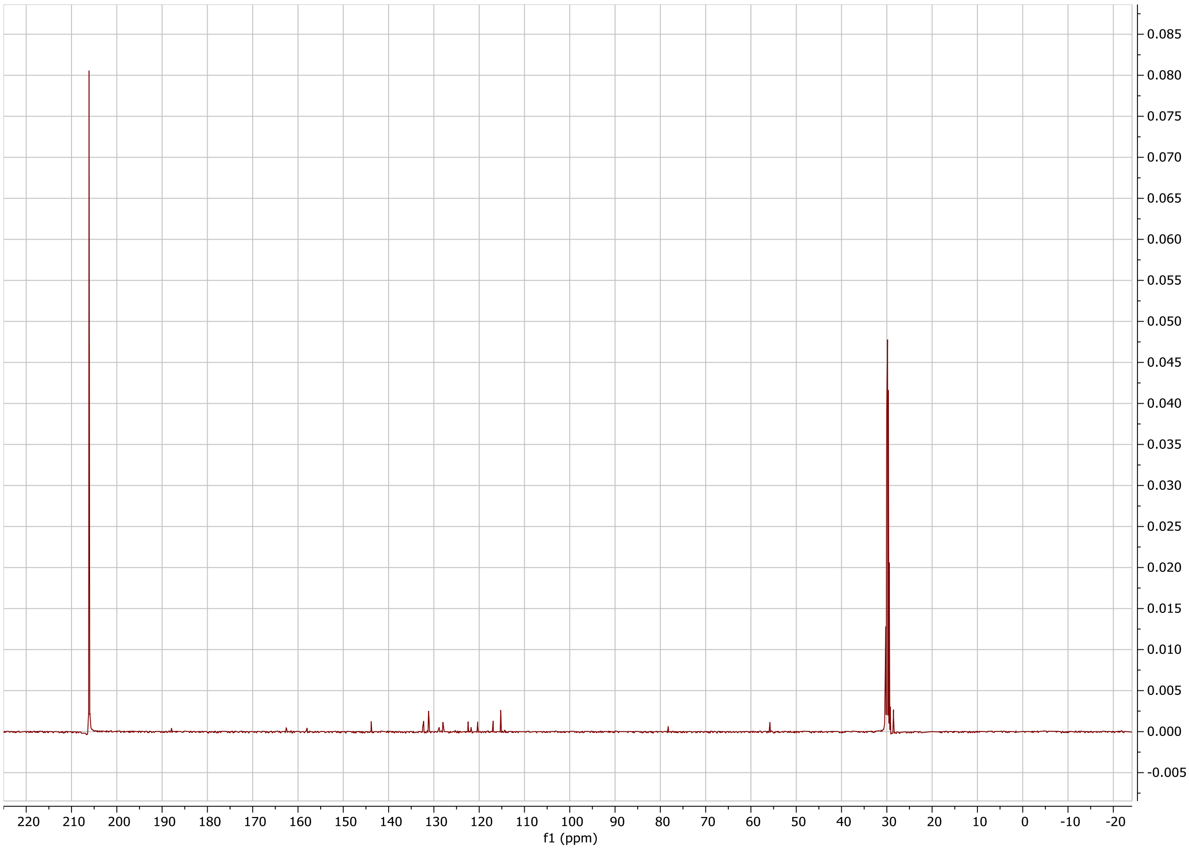

^{1}H and ^{13}C-NMR of compound 11

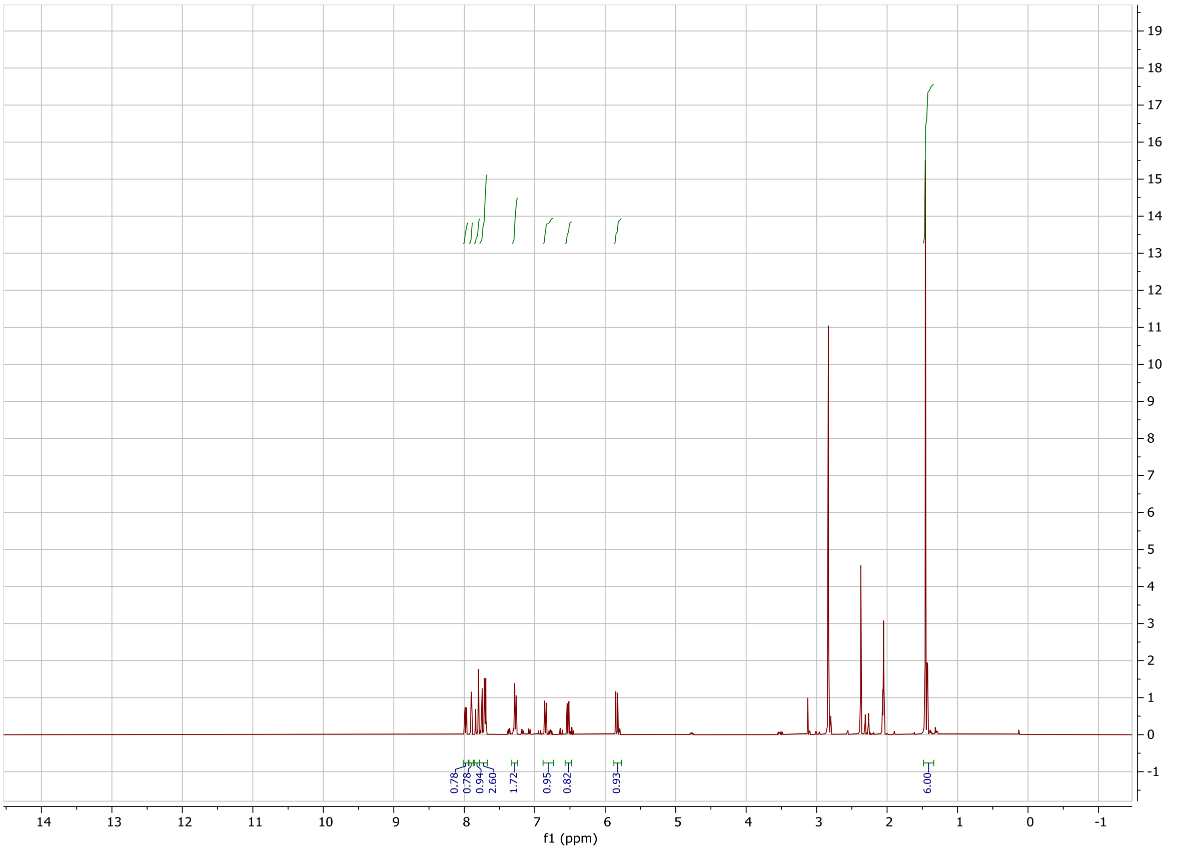

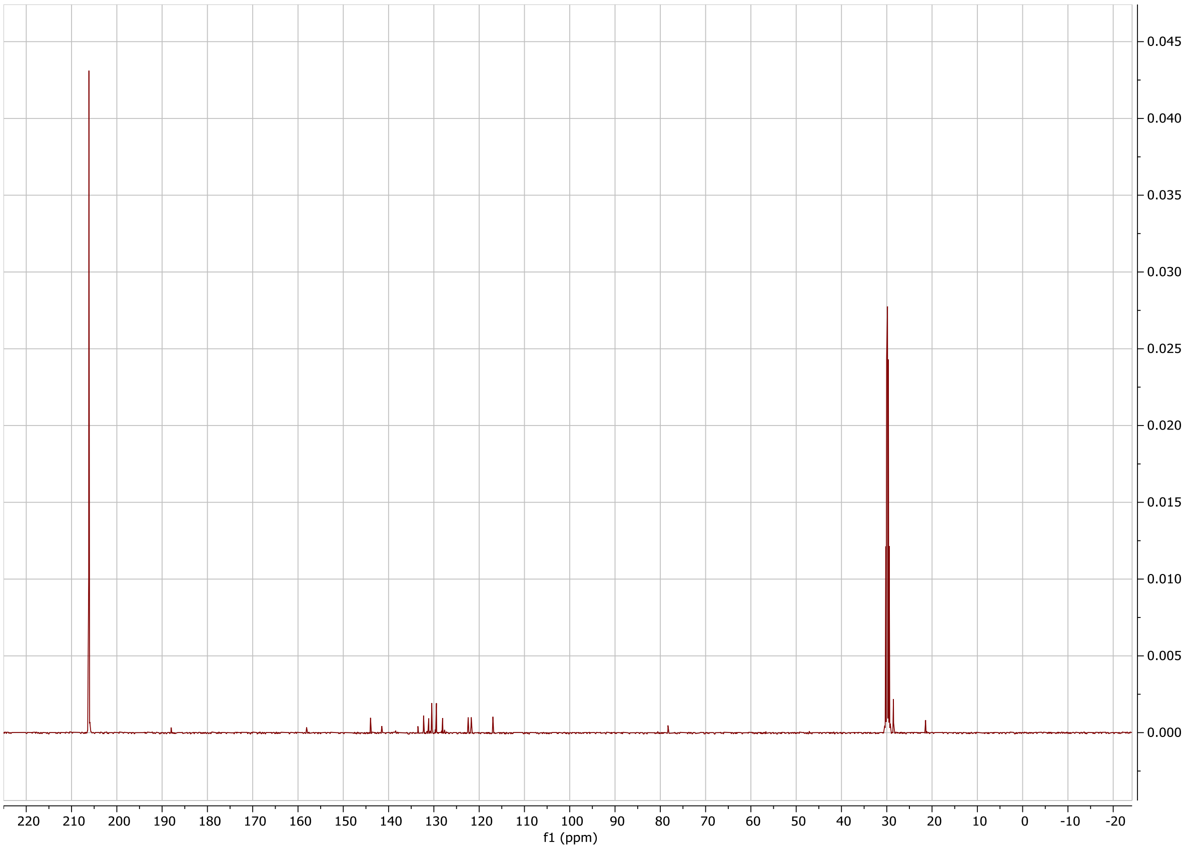

^{1}H and ^{13}C-NMR of compound 13

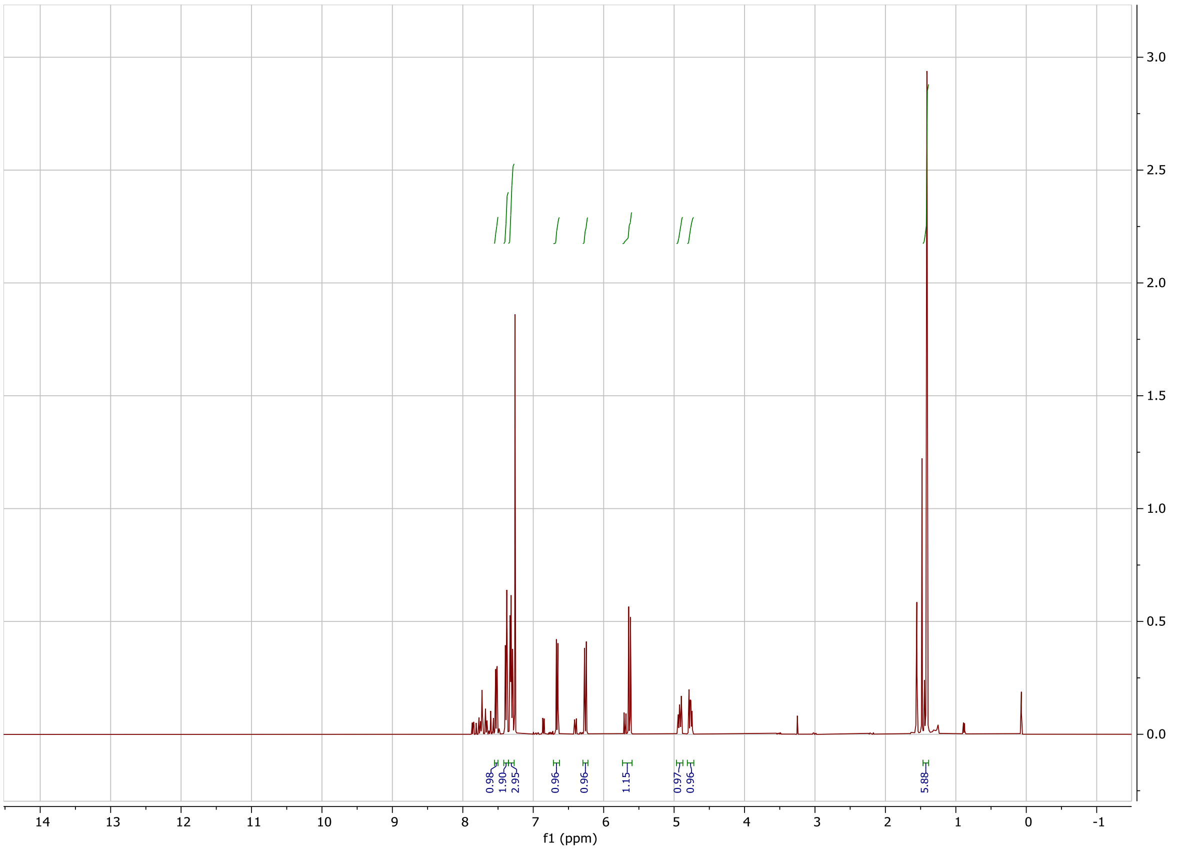

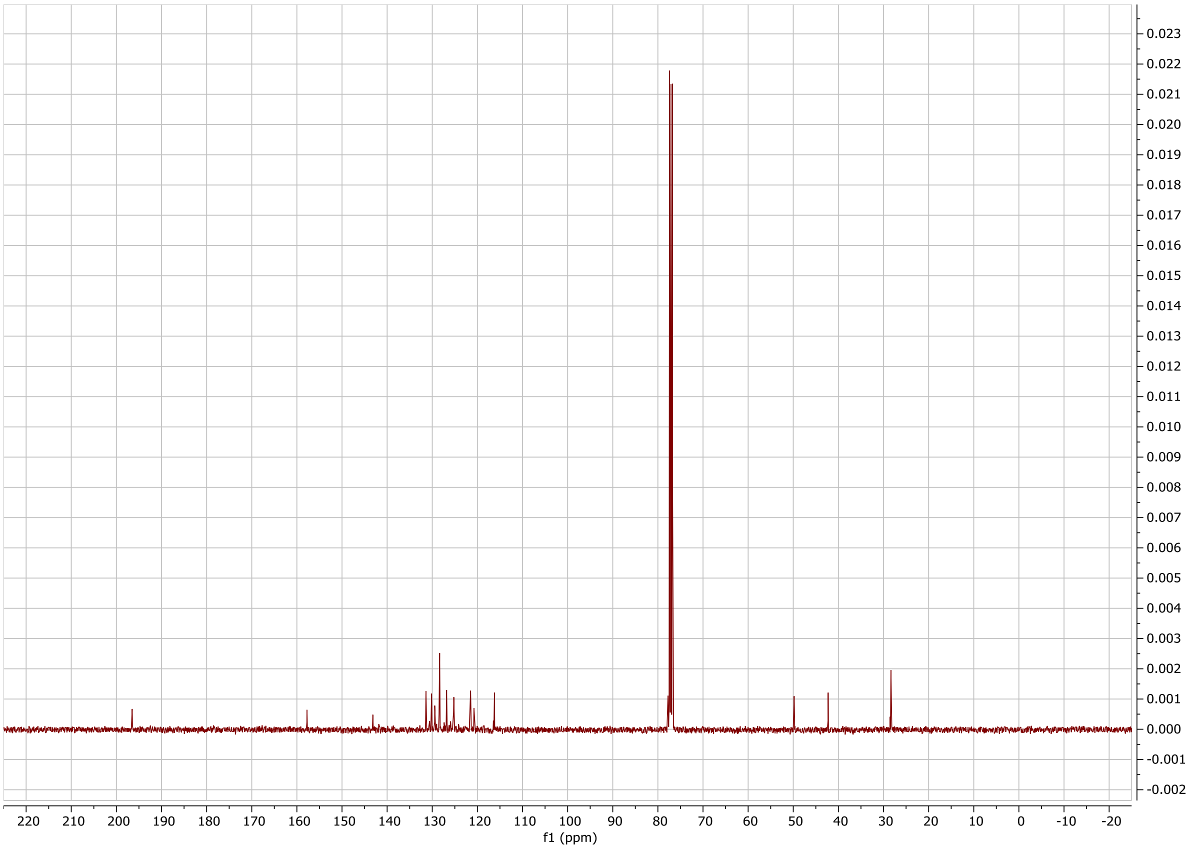

^{1}H and ^{13}C-NMR of compound 14

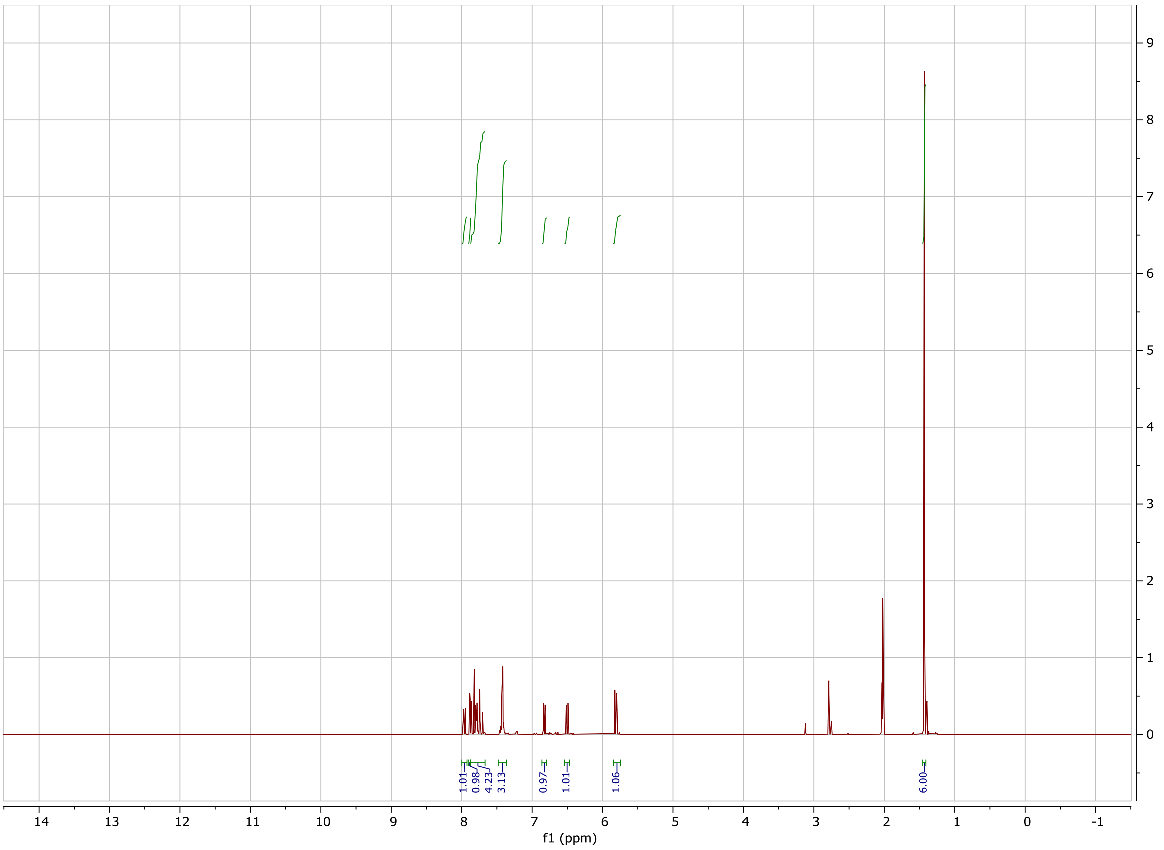

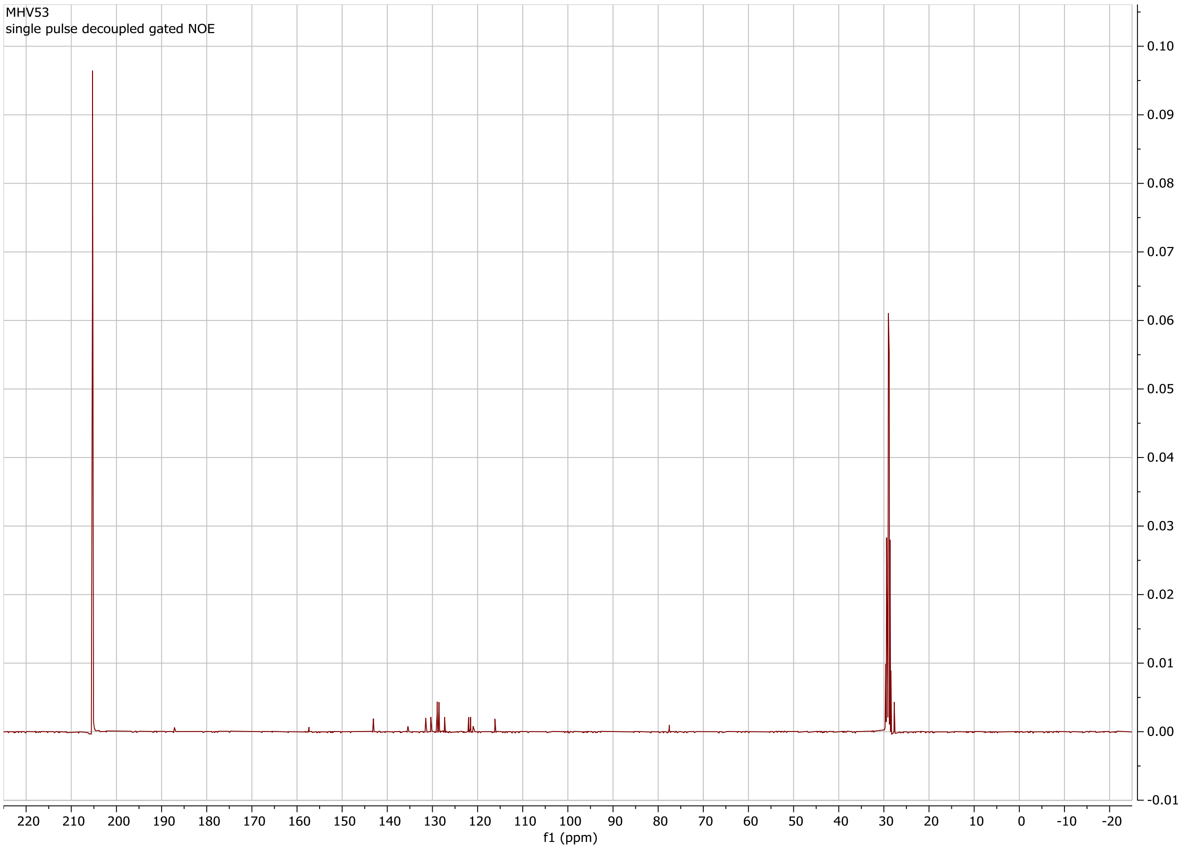

^{1}H and ^{13}C-NMR of compound 15

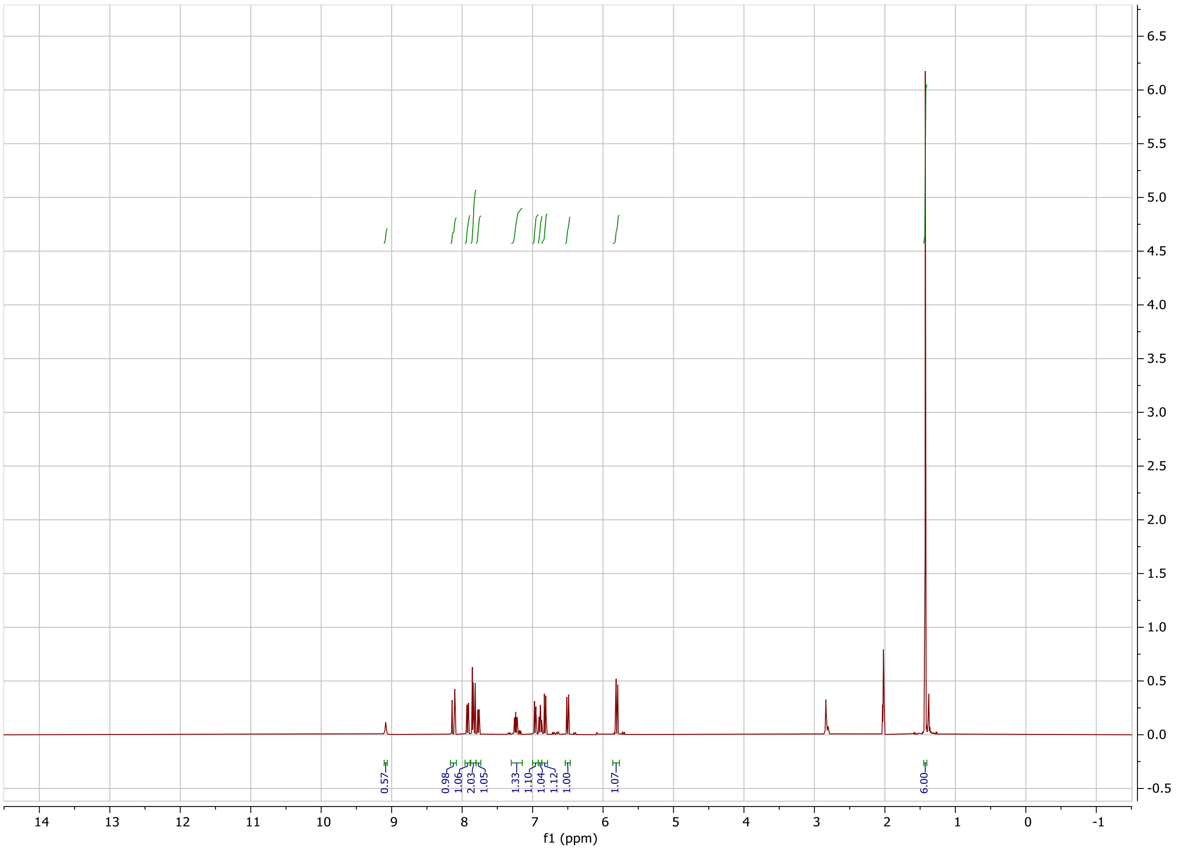

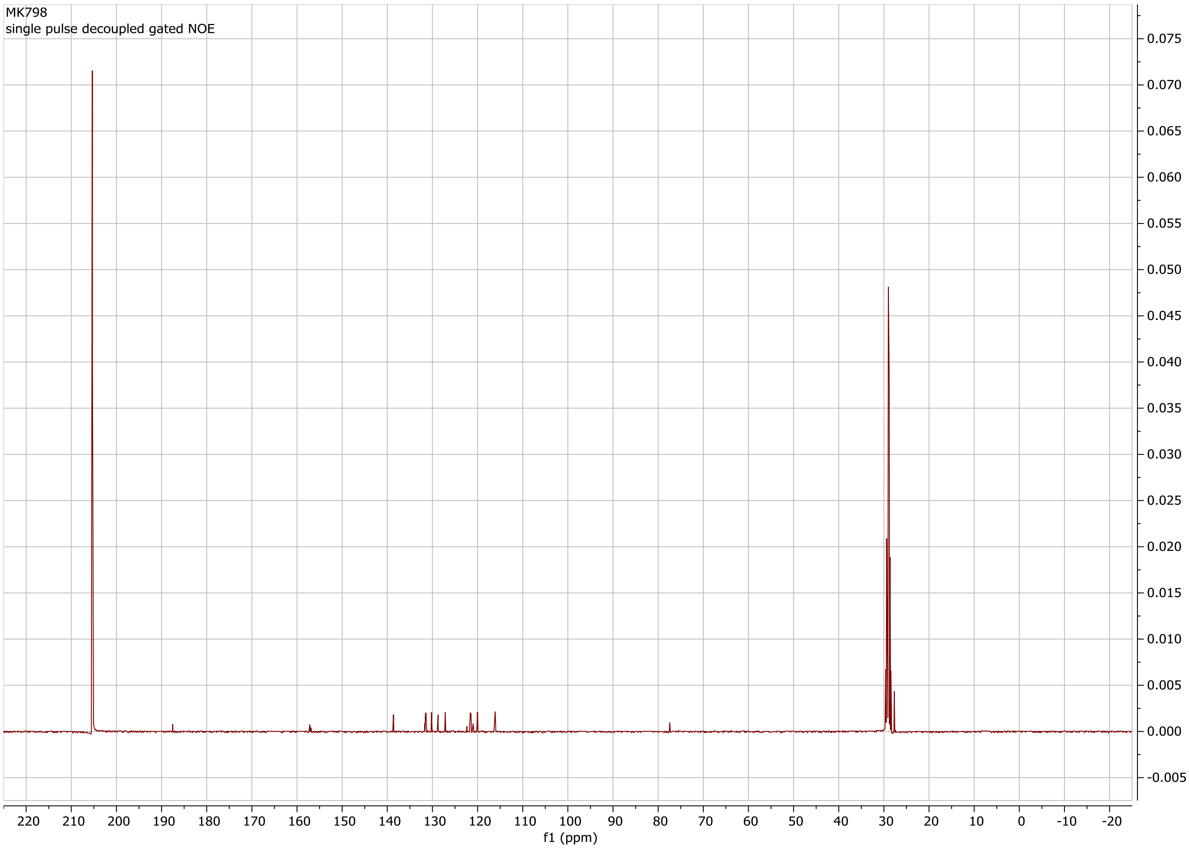

^1H and ^{13}C-NMR of compound 16

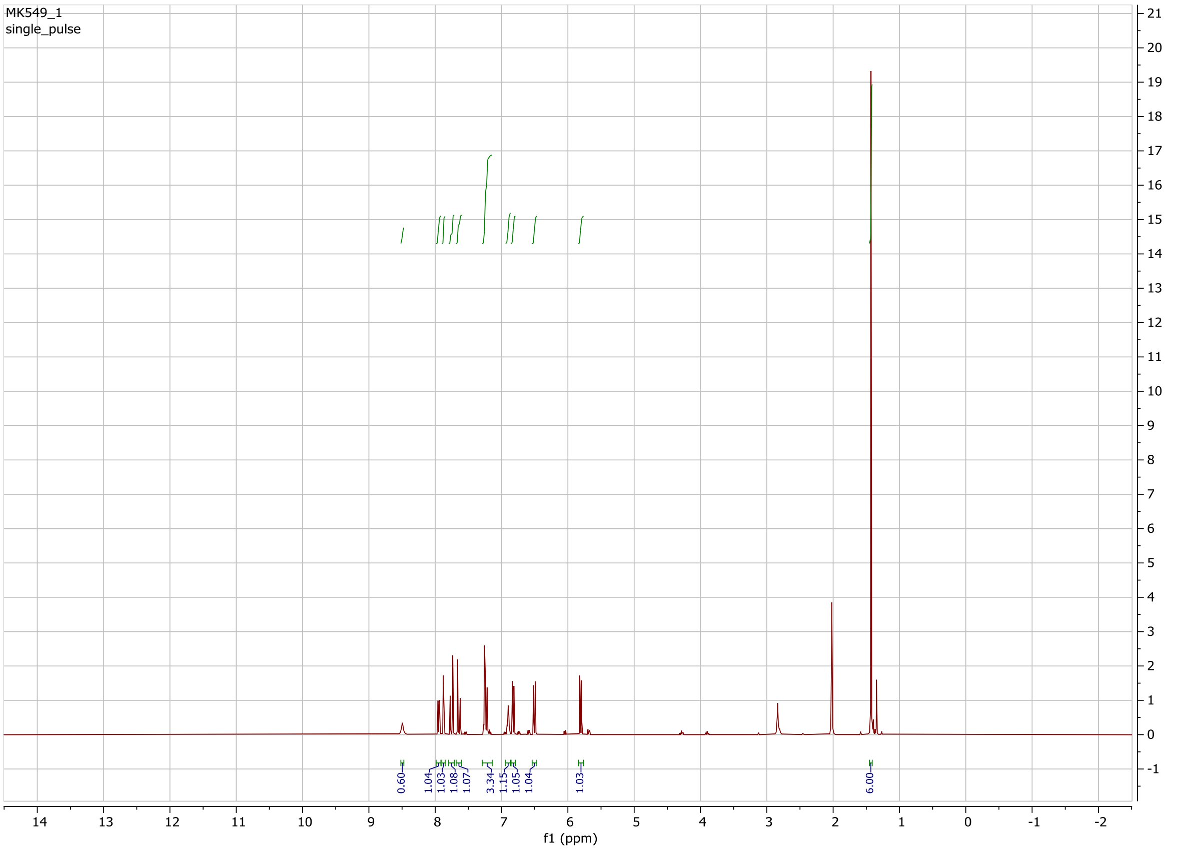

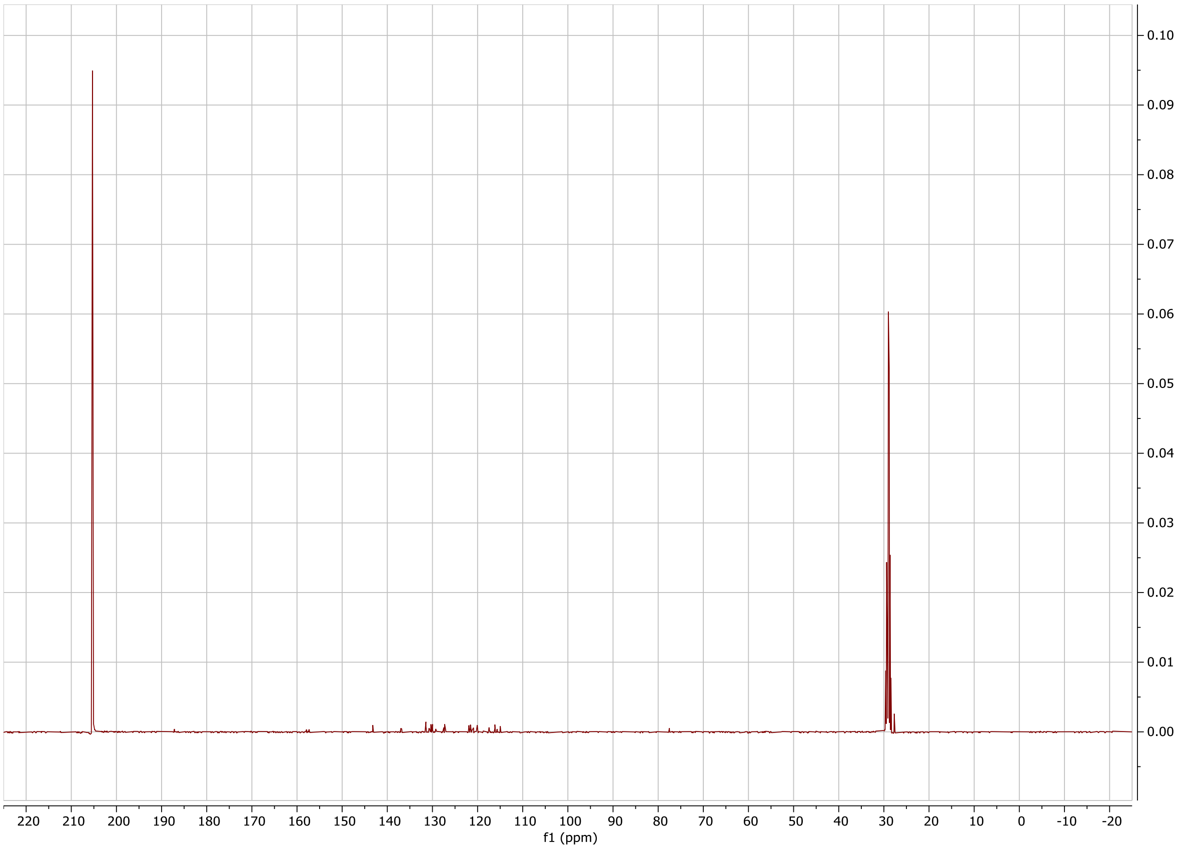

^{1}H and ^{13}C-NMR of compound 17

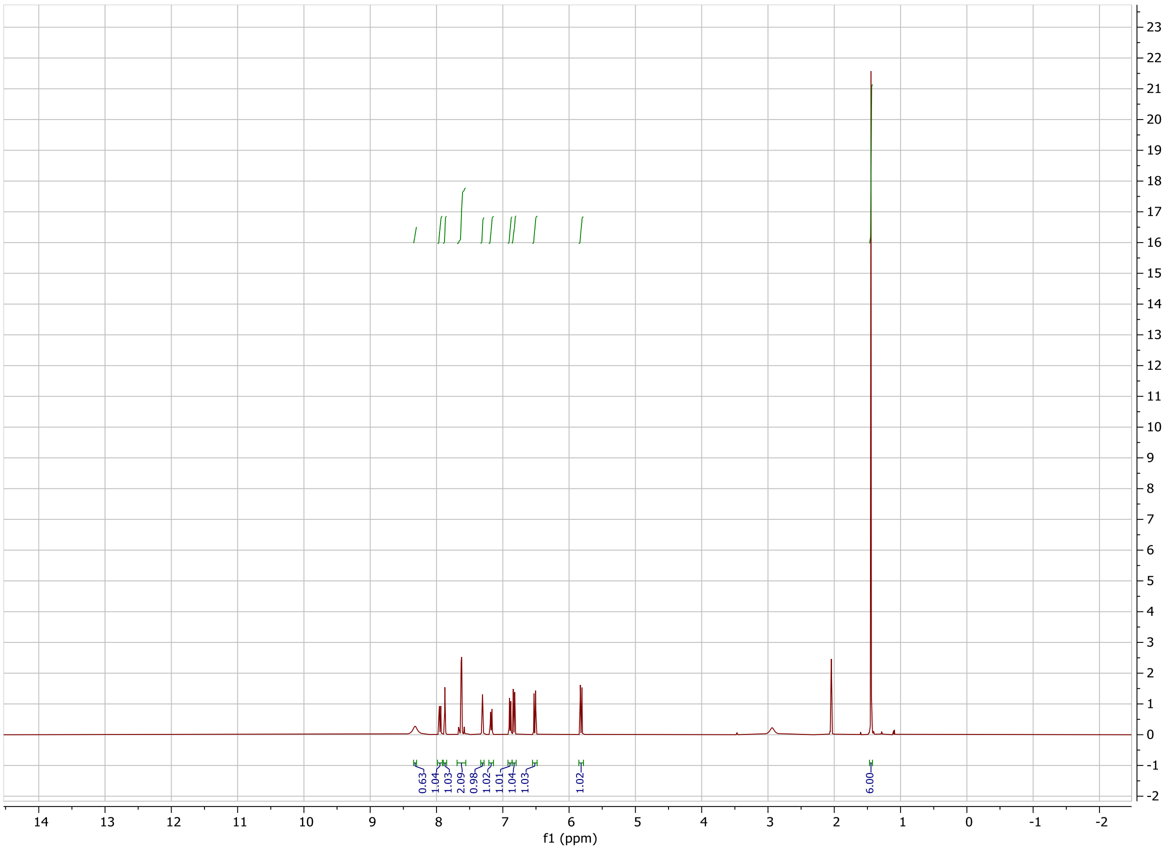

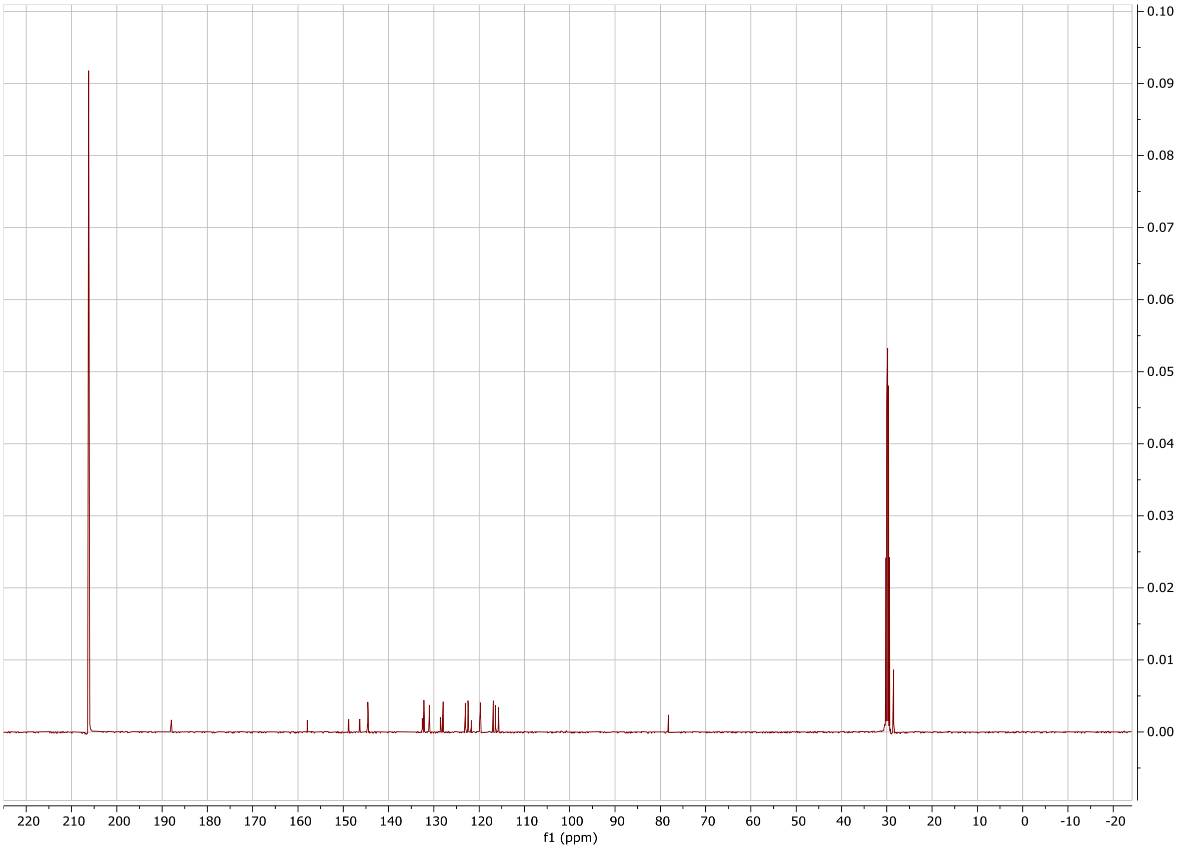

^{1}H and ^{13}C-NMR of compound 18

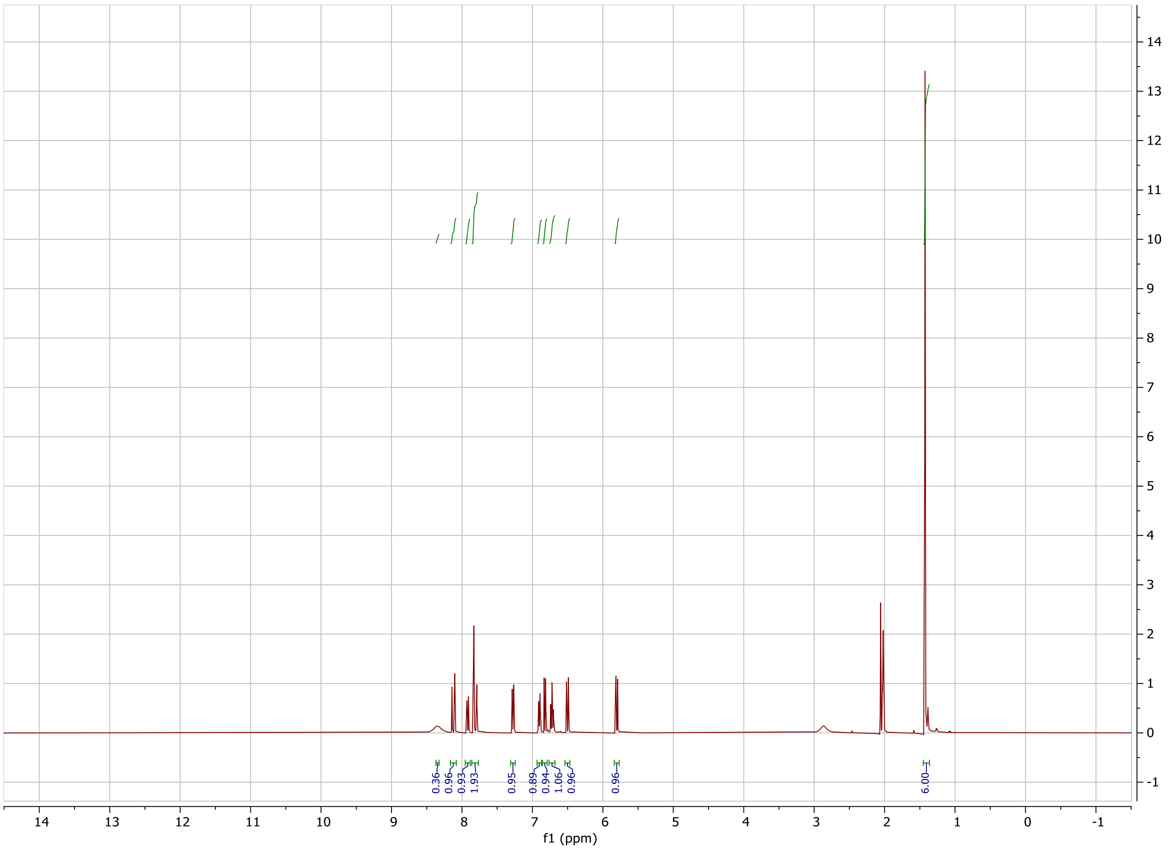

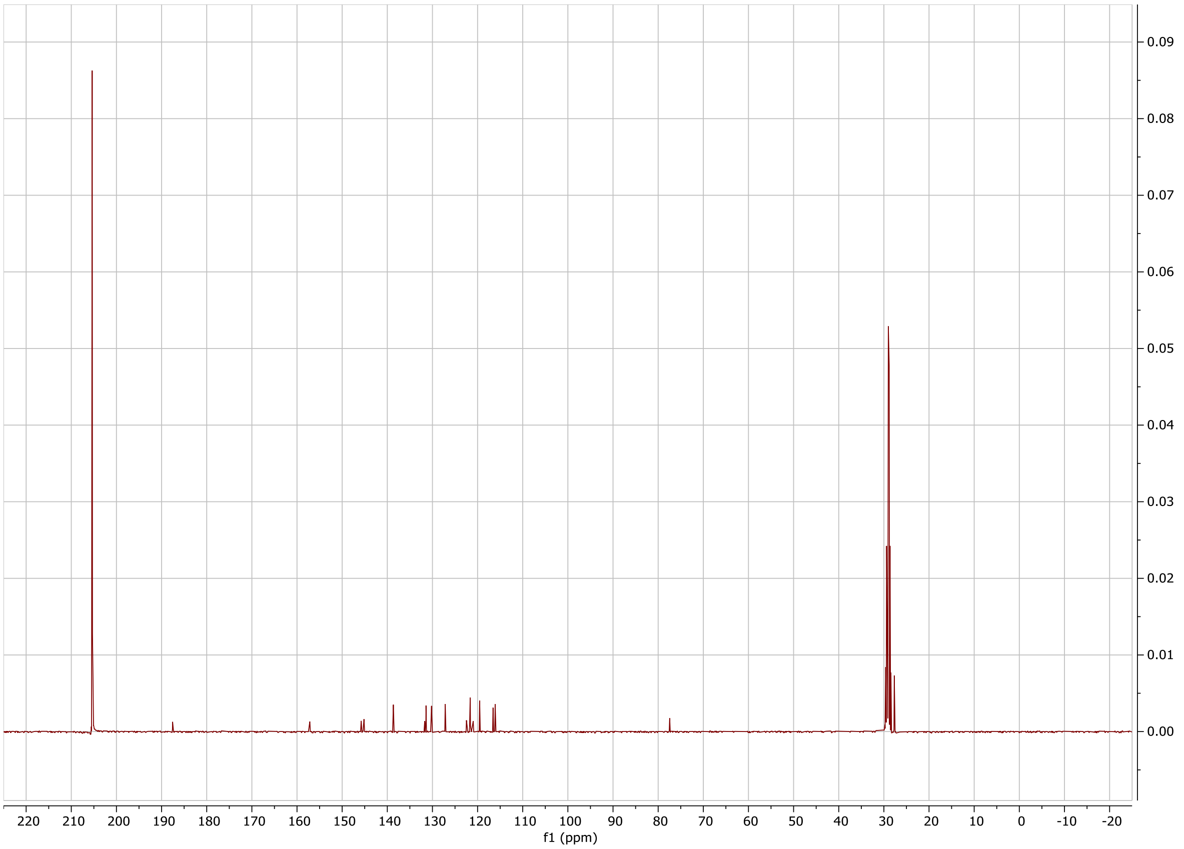

^{1}H and ^{13}C-NMR of compound 19

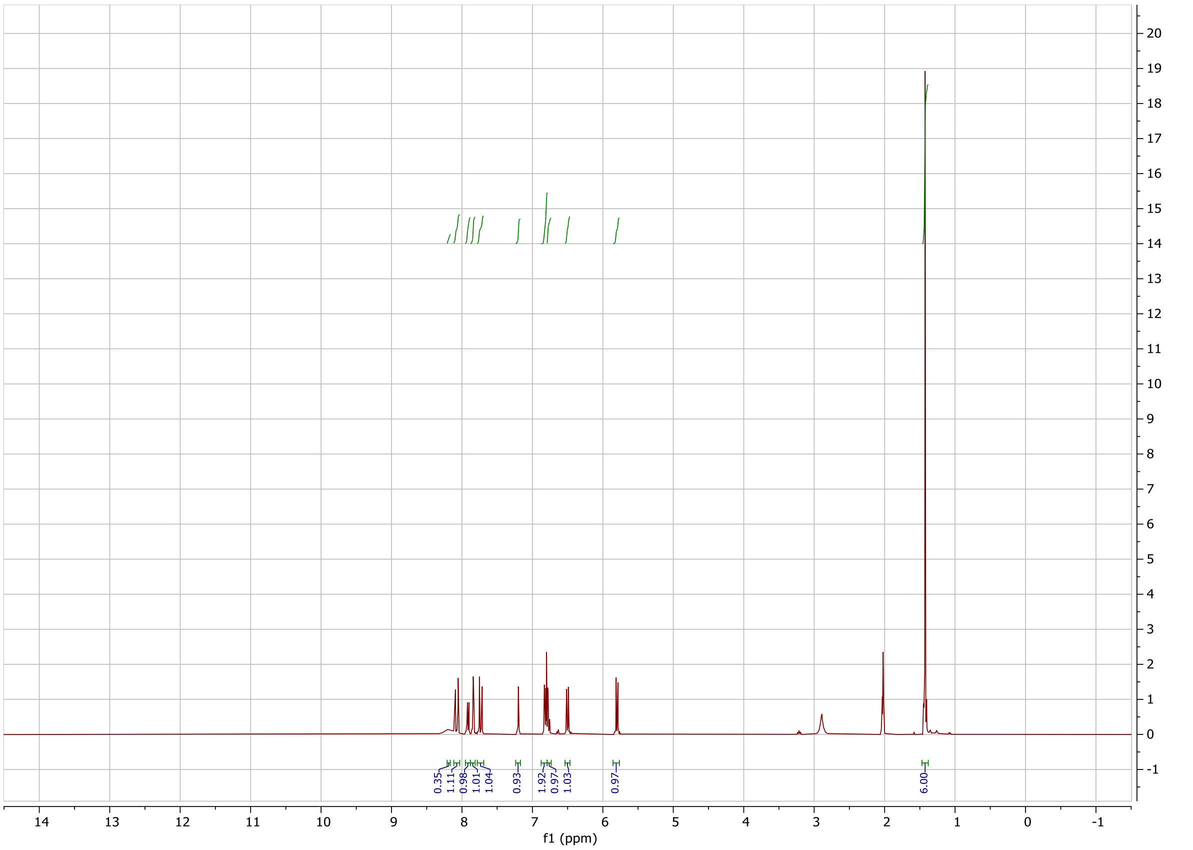

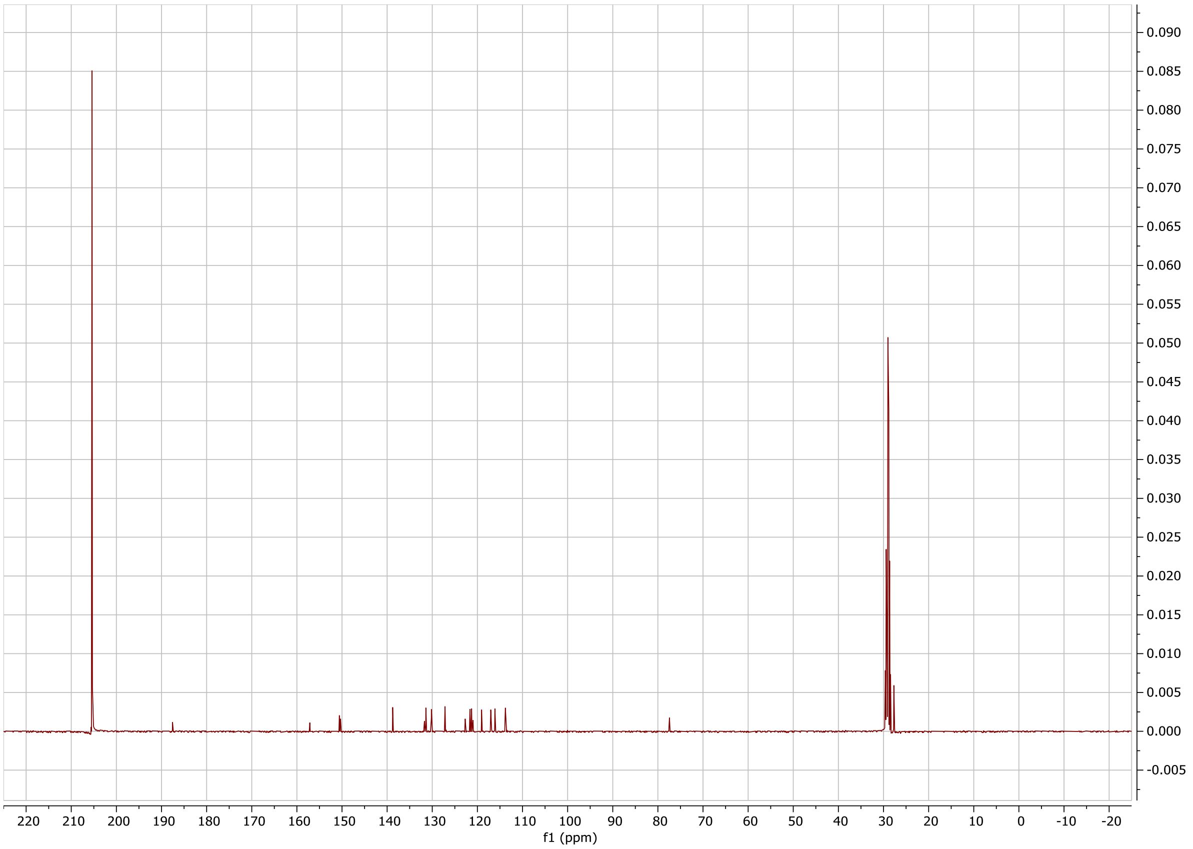

^{1}H and ^{13}C-NMR of compound 20

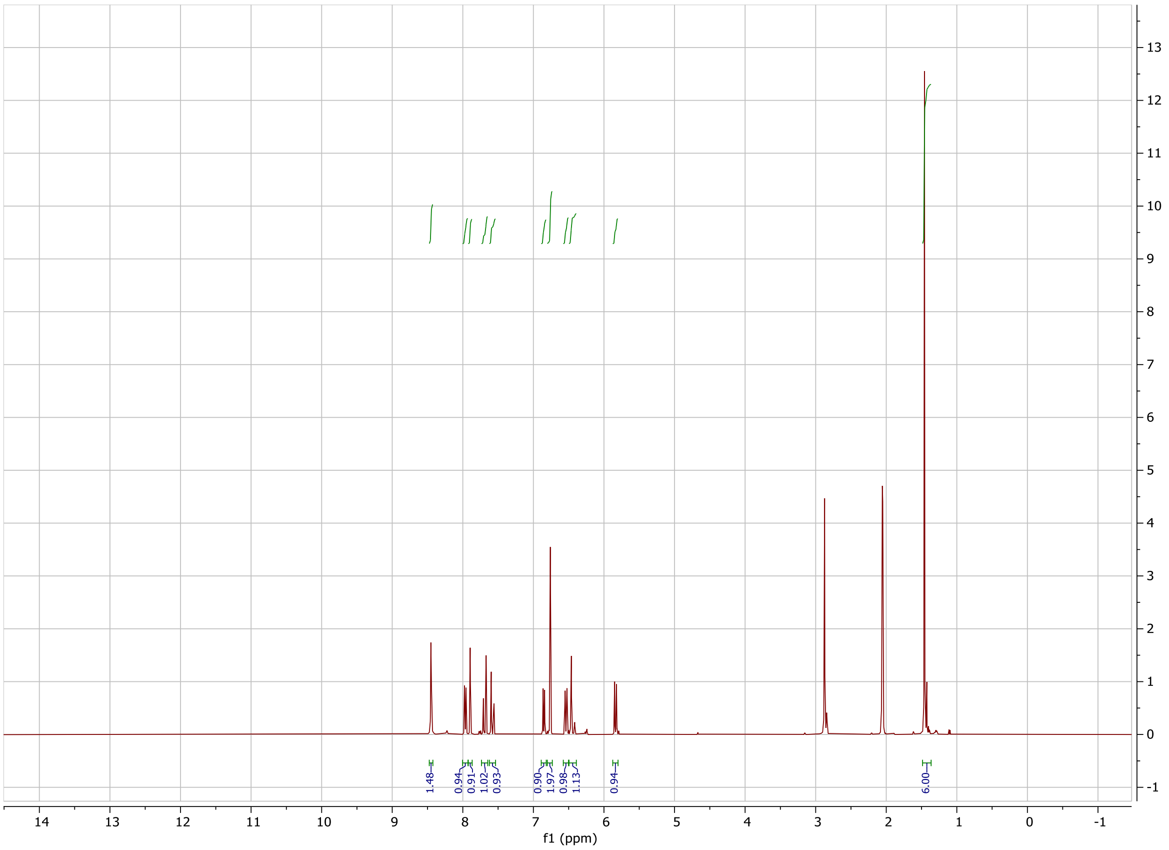

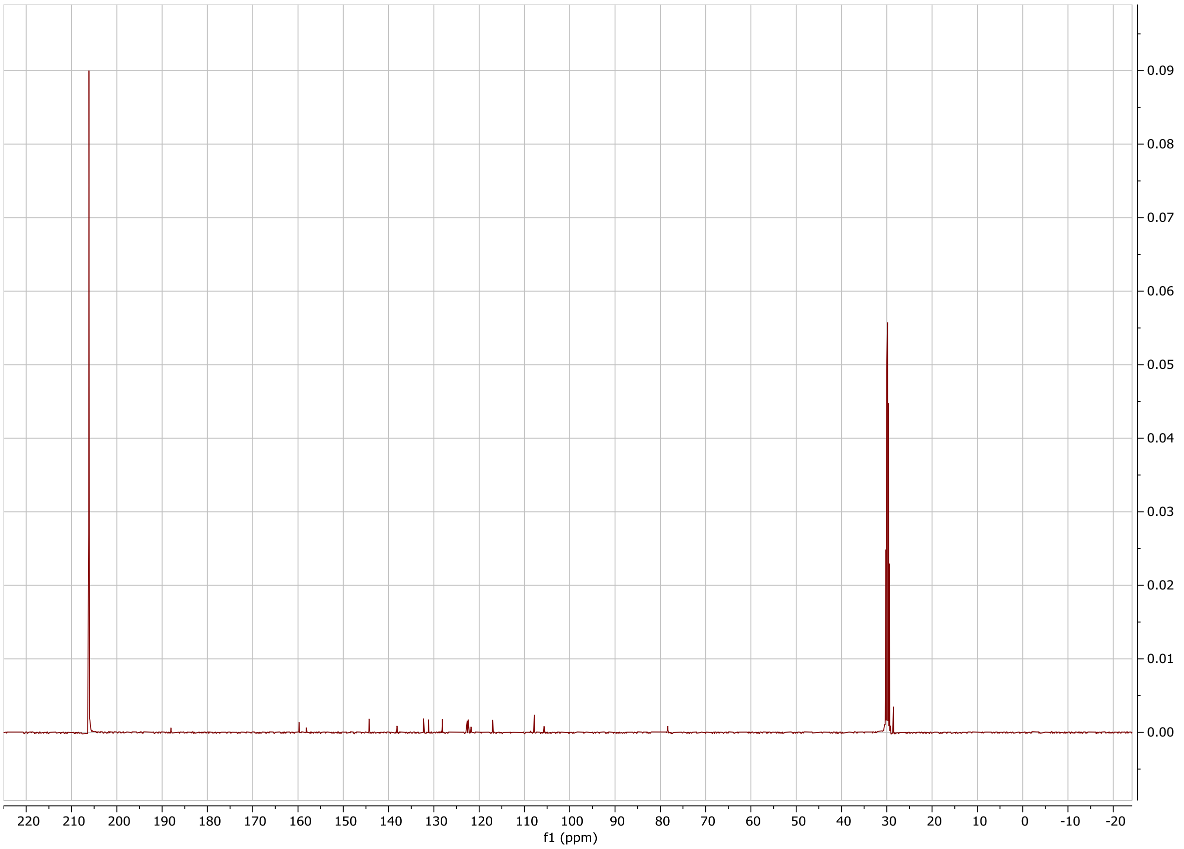

Supplement: 20250417_Supplementary Information wo authors.pdf [file IENZ_A_2509657_SM5270.pdf]
